# Ethnopharmacological Survey of Plants Used in the Traditional Treatment of Gastrointestinal Pain, Inflammation and Diarrhea in Africa: Future Perspectives for Integration into Modern Medicine

**DOI:** 10.3390/ani3010158

**Published:** 2013-03-04

**Authors:** Timo D. Stark, Dorah J. Mtui, Onesmo B. Balemba

**Affiliations:** 1 Lehrstuhl für Lebensmittelchemie und Molekulare Sensorik, Technische Universität München, Lise-Meitner Str. 34, D-85354, Freising, Germany; E-Mail: timo.stark@tum.de; 2 Department of Biological Sciences, University of Idaho, Moscow, ID 83844, USA; E-Mail: dorahmtui@hotmail.com

**Keywords:** folk medicine, alternate medicine, analgesia, cramping, diarrhea, colic

## Abstract

**Simple Summary:**

This review provides an inventory of numerous plant species used as traditional remedies for pain and diarrhea in Africa. Africa can emulate advances in traditional Chinese medicine through research, commercialization, teaching traditional medicine in medical schools, and incorporating botanical products in treating veterinary and human patients. Prioritized research of plant species with proven folklore in treating pain and diarrhea using high throughput screening to identify and test bioactive compounds to verify their effectiveness, mechanisms of action and safety and translational research are needed to facilitate these advances and the integration of traditional African botanical preparations for treating pain and gastrointestinal disorders into western medicine.

**Abstract:**

There is a growing need to find the most appropriate and effective treatment options for a variety of painful syndromes, including conditions affecting the gastrointestinal tract, for treating both veterinary and human patients. The most successful regimen may come through integrated therapies including combining current and novel western drugs with acupuncture and botanical therapies or their derivatives. There is an extensive history and use of plants in African traditional medicine. In this review, we have highlighted botanical remedies used for treatment of pain, diarrheas and inflammation in traditional veterinary and human health care in Africa. These preparations are promising sources of new compounds comprised of flavonoids, bioflavanones, xanthones, terpenoids, sterols and glycosides as well as compound formulas and supplements for future use in multimodal treatment approaches to chronic pain, gastrointestinal disorders and inflammation. The advancement of plant therapies and their derivative compounds will require the identification and validation of compounds having specific anti-nociceptive neuromodulatory and/or anti-inflammatory effects. In particular, there is need for the identification of the presence of compounds that affect purinergic, GABA, glutamate, TRP, opioid and cannabinoid receptors, serotonergic and chloride channel systems through bioactivity-guided, high-throughput screening and biotesting. This will create new frontiers for obtaining novel compounds and herbal supplements to relieve pain and gastrointestinal disorders, and suppress inflammation.

## 1. Introduction

Traditional African herbal medicine (TAHM) is among the most ancient natural therapies and perhaps the oldest folk medicine currently practiced [1,2]. According to the World Health Organization [3], traditional medicine includes “health practices, approaches, knowledge and beliefs incorporating plant, animal and mineral based medicines, spiritual therapies, manual techniques and exercises, applied singularly or in combination to treat, diagnose and prevent illnesses or maintain well-being”. Traditional African medicine is principally based on using botanical preparations to treat animal and human illnesses. In TAHM, medications are prepared by extracting components from entire plants, roots, barks, leaves, flowers, seeds, and aerial parts from a particular herb or plant species as individual entities, or different herb or plant parts of different species combined, or mixtures of extracts combined. Extracts are prepared in the form of decoctions and concoctions, infusions for oral consumptions, enemas and inhalations, or as paste for topical applications on surface lesions including painful swellings and fractures [4,5,6]. Traditional healers mix different ingredients, and in some cases, alter dosage depending on the severity of the illness. For the most part, water is the main medium for the extraction. Ethanol and other organic solvents are rarely used, however, it is not uncommon for herbalists to prepare extracts using local brews. Semi-refined and highly refined preparations, similar to compound formulas used in traditional Chinese medicine [7,8], have not been reported. Typically, in TAHM, animal patients are treated at home or in grazing areas.

Traditional medications and medical techniques are passed down verbally through generations. In most cases the effective or doses and combinations proposed by traditional healers differ; as such the effective doses are not fully known, nor is the effectiveness, safety, toxicity, and variation of chemical composition between plant parts [9,10,11]. Ethnoveterinary medicine observation based evidence and clinical trials are lacking or have not been documented. There are also no reports to suggest the use of traditional herbal extracts in animal clinics. As a result of the lack of scientific facts, TAHM is regarded with much skepticism. The central questions are always: do herbal extracts work? If they do, what are the ingredients/how do they work? And finally, what then should be done to utilize these vast natural resources to benefit animal and human patients and integrate TAHM into western medicine given the skepticism and limited resources? Several publications address this matter in the context of health, policy, social/conservation, and ethical/legal issues [9,10,12]. This review tackles what needs to be done to advance TAHM remedies for the treatment of pain and gastrointestinal disorders (in a general sense), to generate products for integration with acupuncture and western medicine by highlighting what needs to be done in extraction, biotesting, and manufacturing. As indicated for some plant species in this review, numerous African and Chinese medicinal botanical preparations have bioactivity against pain and diarrhea. Therefore, we have included summaries of the plant species used to treat pain, colic, diarrheal, and dysentery based on documented anecdotal treatment practices and in some cases, scientific evidence from literature (Table 1 and Table 2). The goal is to provide a broader outlook, which is needed to form the basis for selecting species for biotesting and the development of effective medications for indigenous use, and the integration as adjunctive therapy into acupuncture and western medicine. The list highlights plants with potential for new medications that can be used to improve the treatment outcome and quality of life for patients suffering from gastrointestinal disorders and the accompanying pains. Most medicinal plants are used to treat more than one disease or disease symptoms, therefore, it’s possible that this wealth of herb and plant species can be exploited for novel therapies against traumatic injuries, neurological disorders, cardiovascular, renal disease, or other illnesses of veterinary patients. 

There is evidence to suggest that folk formulations of plant, animal, and mineral origin are widely used to treat veterinary patients and, to some degree, for the improvement of livestock production [9,13]. Nonetheless, the role and contribution of ethnoveterinary medicine in animal health and production in Africa are not fully known. In contrast, 80% of people in Africa and 65% of the world population depend on folk medicine for primary health care and each year over US $ 83 billion is spent on traditional alternative, or complementary medicine [14]. There is more information in the literature concerning plant species and extracts used to treat human patients compared with ethnoveterinary practices in Africa. It has also been found that the types of plant species used to extract medications for animal and human healthcare overlap significantly [9]. Clearly, the identification of the most suitable plant species for treating pain and gastrointestinal disorders in animal patients requires a good understanding of plant species used as treatments against these conditions in human patients. This goal of identifying novel medications for gastrointestinal disorders and pain could benefit from testing fractions/compounds isolated from traditional extracts used to treat chronic pain and inflammation such as arthritis, particularly in cases where related plant species are traditonally used to treat gastrointestinal ailments. 

The theme of this Special Issue is the “Combination of Western and Chinese Medicine in Veterinary Science”. While traditional Chinese medicine is receiving broader recognition in the world, including integration into western treatments, skepticism about the effectiveness and lack of information about bioactive components, and the mechanisms of action and safety of TAHM are key limiting factors for the integration of TAHM into evidence-based western medicine for the treatment of pain, trauma, and gastrointestinal issues in veterinary and human patients. There is no doubt that Africa has a wealth of diverse plants species, each with a high potential for medicinal value that fit into this niche, but have yet to be fully explored. In support of this notion, we provide an extensive, yet not comprehensive, inventory of plant species and parts used to extract medications for the treatment of pain and gastrointestinal ailments—especially colic, diarrhea, and dysentery—in this review (Table 1 and Table 2). As summarized in the inventories, there is limited scientific evidence to verify if TAHM remedies are effective against illnesses documented through folklore, and if they are useful in the improvement of animal and human health. Overall, studies to authenticate anecdotal knowledge have shown the effectiveness of TAHM preparations against pain, colic, and diarrheas. Bioactive components have been identified for some species, in particular, the class of compounds found in extracts. With more research, some of the traditional treatments have potential to be accepted and incorporated into modern animal, and human, medical practices. They may also provide the stimulus to identify new bioactive compounds for production of mainstream (evidence-based) pharmaceutical medicine.

## 2. The Potential for New Drugs to Treat Pain and Gastrointestinal Disorders from TAHM

### 2.1. Resources Available from Indigenous Knowledge and Literature

Infectious diarrhea, dysentery and chronic diarrheas, and colic are severe, debilitating conditions presenting with abdominal pain and in some cases inflammation. Acute visceral pain involves the activation of high-threshold nociceptive fibers, while chronic visceral pain is thought to be due to sensitization of both extrinsic and intrinsic mechanoreceptors by conditions such as inflammation or ischemia [15,16,17]. Therefore, effective herbal remedies of pain and diarrheas are likely to contain neural active compounds and in the case of chronic pain remedies, may have anti-inflammatory components. 

The need for medications that are used to treat patients with chronic gastrointestinal illnesses presenting with pain and inflammation is unmet and growing. For thousands of years, botanical medications have played a significant role in public health as treatments [1,2,9,14,18,19] and sources of new drugs and will continue to do so [20,21]. Therapeutic uses of about 80% of 122 plant-derived drugs correspond to their original ethnopharmacological role [20]. This suggests that careful screening of the diverse botanical preparations used in TAHM—or derivative fractions and compounds—have a potential for new therapies against painful, chronic gastrointestinal disorders, and integration into western medical treatment practices of veterinary and human patients. One example, and perhaps the best fit for this theme, is the root extract from *Harpagophytum procumbens*—a plant native to Saharan Africa—that has been accepted for use (together with acupuncture) as complementary treatments for osteoarthritis in western veterinary and human medicine. The basis for the acceptance of this plant product, and its effectiveness, is in containing components with analgesic and anti-inflammatory properties (such as triterpenoid glycoside beta-sitosterol) and the known side effects of lowering blood sugar, and increasing stomach acids and rhythmic cardiac activity [22]. Better complementary drugs are needed and African botanical folk therapies offer the most valuable resource. In the Table 1 and Table 2, plant species with the dagger sign after their names appear to be the most widely used in TAHM against painful gastrointestinal illnesses and diarrhea. Most of these plants are also used to treat several chronic illnesses (not included) suggesting that they should be given prioritized research to support and advance their therapeutic uses.

Medicinal plants used to treat human pain and diarrhea are covered here because plant species used to extract products for treating animal and human pain and diarrheas overlap. Roughly, over 150 medicinal plant species are used to treat painful gastrointestinal illnesses (58 families), diarrhea, and dysentery in animal patients in Africa (Table 1). By contrast, there are over 250 medicinal plant species (75 families) used for similar purposes in TAHM to treat human patients (Table 2). A summary of plant families in Table 3 indicate that leguminosae, compositae, lamiaceae, rubiciae, anacardiaceae, malvaceae, menispermaceae, and apocynaceae are the major medicinal plant families for both animal and human use in Africa, with over 70 plant species being used to treat both veterinary and human patients (Table 1; family name with asterisk sign). The overlap shown here emphasizes the need for veterinary investigators/practitioners to become familiar with the plant species used to treat similar or related human illnesses, whether it is in Africa or other parts in the world. Families that appear to be prominent sources of plant medications for humans but not animals are clusiaceae, aizoaceae, capparaceae, convolvulaceae, hypoxidaceae, lauraceae, and salicaceae. Compared with Africa (Table 1 and Table 2), the most common Chinese ethnoveterinary plant products are from the family ranunculaceae (Table 4), followed by papaveraceae and leguminosae (Table 3). Collectively, summary data supports the notion that herbal medications for human illnesses have been more extensively studied than botanical preparations used for treating similar conditions in veterinary patients. What is alarming is the lack of information found in the literature about the treatment of acute/chronic pain, diarrhea and dysentery in cats and dogs. 

Although, the African Herbal Pharmacopoeia has been published [2], it is only covers a few selected plant species. The available databases on African medicinal plants, such as: http://www.ulb.ac.be/sciences/bota/pharmel.htm, http://www.prota.org/PROTAstartframes.htm and http://www.ippc.orst.edu/ipmafrica/db/index.html are not adequate. There is need for more TAHM electronic databases such as monographs of plants and plant derivatives used in veterinary and human alternative medicine practices in Africa. Such resources of summary data will be valuable to research, teaching TAHM, and public knowledge and could be made available through the Pan-African Natural Products Library.

In the past two decades significant progress has been made in research on TAHM, as indicated by the number of publications with South African institutions taking a lead. Subsequently, the majority of plant species described here are from Southern Africa, Nigeria, Egypt and East Africa. This indicates that more work is needed to identify and/or document plant species used to treat veterinary and human pain, diarrhea from other areas of Africa to fully exploit the indigenous herbal medicine in Africa. We, and other scientists [23], have observed that there are several gaps and inconsistencies in gathering information about herbal African folklore medicine. Particularly interesting are inconsistencies found in the documentation of what parts and exact ratios of plants are suitable for a particular medication or, whether the plant preparation is used as an individual or in a mixture [4,24,25]. African governments and institutions such as the Natural Products Research Network for Eastern and Central Africa (NAPRECA; [12]) should establish standardized “guidelines” of gathering this data for all researchers in order to produce accurate documentation of indigenous plant use in Africa. 

### 2.2. Strategic Research to Advance TAHM

The advances associated with verification of efficacy, bioactive compounds, safety, and semi-processing to pack and preserve traditional Chinese medications have helped bolster the integration of Chinese medicine into western medicine. Traditional African herbal medicine (TAHM) utilizes organic botanical products, which could contribute to global improvement of animal health care and production, either as individual preparations or as adjunctive therapies to western medicine. However, this will require carefully guided strategic research. Pre-requisites for research priorities are identifying critical needs of global veterinary/human medicine for new or adjunctive therapies to improve health and wellness (in this case, treating pain and gastrointestinal disorders). As shown in this review, many natural medications against body pain and gastrointestinal conditions with symptoms of diarrheas and dysentery are available in Africa. Prioritized research is necessary in order to determine what plant species or derivertives should be analyzed fully. A careful and thorough literature review dealing with the described folklore is needed to identify and select plant species to be considered for research priority. Additional considerations include: comparison of existing information with literature about patient/farm-evidence based social studies, laboratory tests, and clinical/field trials across the world [11,26,27]. National and pan African advisory councils for complementary and alternative medicine in veterinary care should lead the prioritization and botanical drug development initiatives for integration or adjunctive therapy with acupuncture and western medicine.

### 2.3. Needs in Basic Research

Empirical approaches for scientific validation of the efficacy, safety, bioactive components, and mechanisms of action are necessary for the progress towards integration of TAHM into western medicine [7,26,27,28]. Systematic, prioritized research that provides irrefutable, proof-of-principle evidence for interventions against pain and gastrointestinal disorders described in folklore practices are necessary prerequisites to bolster the integration of TAHM into modern medicine. Strategic, basic research should focus on biological effects and mechanisms of action underlying the described folklore practices, and characterize the active components of the intervention. Verification of cellular mechanism should use *in vivo* and *in vitro* methods by means of simple methods for fast screening, if possible, accompanied by state-of-the-art techniques to demonstrate and quantify bioactivity. Most medicinal herbs/plants have several medicinal compounds (or compounds of similar classes) that exhibit similar medicinal effects. Determination of classes of compounds can be done using simple chemical tests and is useful to validate traditional use. These tests should not be employed as screens that provide justifications for traditional uses only [23]. Instead, testing should target the isolation, characterization, testing of bioactive components and establishing optimal doses, non-specific *vs.* specific effects, bioavailability, side effects, and toxicity levels. 

Research should not be viewed as a means to discover new compounds, but rather, should be seen as an absolute need to develop the most promising plant therapies into either unmodified natural products (crude extracts), semi-refined products (fractions/sub-fractions or recombinants of fractions), and/or pure compounds and compound formulas [4,8,28,29,30]. Semi-processed products can be cheaper and have concentrated active components without constituents with adverse or toxic effects. They then become best suited in quality, efficiency, safety, and affordability for use as medications or diet supplements in the treatment of veterinary and human patients [8,29,31]. To achieve this goal, research should be prioritized toward selected plant species, and information about processing, packaging, stability, quality, dosing, preservation of natural habitats, and farming selected plants should be gathered though research. 

### 2.4. Technical Considerations in Screening Herbal and Plant Extracts for Bioactivity and Development for Therapeutic Uses

Screening of bioactive ingredient/s from natural products such as plant materials is best achieved through bioassay-guided fractionation. Generally, a bioassay is any *in vitro* or/and *in vivo* or *ex vivo* system used to detect the biological activity (effects) of a crude extract, fraction, or a pure isolate/compound from a living organism. Biotesting might be done using isolated cells, organs, tissue preparations, animals or human subjects. Combining the separation/isolation and characterization of bioactive ingredients with bioassays is called bioassay-guided fractionation. The extract, fractions, subfractions, and recombinants of fractions and subfractions are screened and tested for bioactivity in natural concentrations within the initial crude extract for bioactivity that correspond to that of the initial crude extract using the same bioassay. The components showing high activity, or higher activity, than others are further bioactivity-guided fractionated until the bioactive compound/s is/are obtained in pure form. Chemical assays—like antioxidative assays (e.g., Oxygen Radical Absorbance Capacity (ORAC) [32] or hydrogen peroxide [33] scavenging assays—are not considered to be bioassays but antioxidative-guided, or in a more general sense, activity-guided fractionation [34]. 

It is absolutely essential to use the fractions and subfractions in their natural concentrations/ratios, to perform recombination and omission experiments for comparing their activity in relation to the activity of the crude extract described through folklore practices. The advantage of using this approach is that chemical changes that occur during extraction, fractionation and isolation, synergistic and/or antagonistic, as well as additive (or a combination of these effects), can be traced. The majority of botanical therapies are extracted using water. Depending on the bioassay and isolation methods, water extraction can be problematic. It requires powerful organic solvents—such as dimethylsulfoxide [35] to be used prior to a final dilution in a buffer in appropriate concentrations for the bioassay. There are other procedures to overcome solubility problems such as using polyvinylpyrrolidone to form soluble complexes [36]. However, compounds like polyphenols, could interfere with the corresponding bioassays (receptor binding and enzyme assays) through nonspecific binding to proteins. These compounds should be removed by appropriate techniques (e.g., pre-separation, precipitation, or adsorbents like XAD-2 resin) but must be developed and tested individually to determine how each impacts the extract and bioassays to ensure that the method itself does not affect the bioactivity, which is cumbersome. Control experiments using blanks, control solutions/samples, as well as positive controls are mandatory during bioactivity-guided screening.

In most cases, the bioactivities of the fractions, subfractions, and pure compounds are lower than that of the initially used extract. This might be the sum of—or individual influence of—losses during workup (e.g., solvent fractionation), different chromatography techniques (e.g., column chromatography), evaporation of fractions, freeze-drying, and photosensitivity. Another possibility is that separating the extract into different fractions or compounds disrupts synergistic and/or additive effects. For instance, we have found that thin layer chromatoghraphic separation of *G. buchananii* stem bark extract yields fractions with anti-motility and pro-motility effects in guinea pig colon [37], suggesting that the crude extract contains compounds with opposite/antagonistic biological effects. In some cases, fractions show greater activity than the crude extract, which might be explained by suppressive and disturbing effects by the other ingredients in the crude extract. However, it is also possible that breakdown products (artifacts) generated during work-up and isolation have stronger activity than the initial (precursor) compound. To overcome these problems light should be excluded during work-up/isolation (brown glass), and inert gas atmosphere (nitrogen or argon) and cooling (ice bath, fridge, freezer) should be used. One approach to prove if artifacts are generated is to use non-targeted nuclear magnetic resonance (NMR) spectroscopy or liquid chromatography mass spectrometry (LC-MS) based metabolomics. Comparing the crude extract with its subfractions could give hints of new compounds generated during isolation/fractionation process. Also, accurate quantitation of the pure bioactive compound by means of LC-MS/MS, NMR or LC-UV in the crude extract, and subfraction followed by comparing the concentrations, could give evidence on breakdown, formation and degradation. 

Another necessary step, after the identification of the bioactive component, is the accurate quantitation, especially if more than one compound was identified, and rating them for their bioactivity on the basis of activity-over-threshold (AoT) (threshold = EC_50_, IC_50_, activity equivalent…) in order to bridge the gap between pure structural chemistry and bioactivity. AoT-factors could be seen as a model approach and representing the individual contribution of each single compound to the activity of the crude extract. The higher the AoT-factor, the higher the contribution a single bioactive molecule has on the bioactivity of the whole extract. With the quantified bioactives the proof-of-principle in form of reconstitution (recombination) and omission experiments should be performed. A reconstitute should be prepared by blending solutions of the individual bioactive compounds in their “natural” concentrations and comparing the bioactivity of this mixture with the crude extract. The goal of such experiments is to prove whether the isolation, purification process, and quantitation steps alter bioactivity. Discrepancy between recombinants (the “cocktail”) and the crude extract suggests bad quantitation during preparation of recombinants, or synergistic compounds are not present in the mixture, and/or important bioactive compounds were altered during separation as mentioned above.

Finally, omission experiments should be performed to demonstrate the importance of the bioactivity of compound classes, or single compounds, in comparison to the whole recombinant in their “natural” concentrations. With this strategy additive, antagonistic, and synergistic effects can be demonstrated. An example for the whole work-flow, bioassay-guided separation and beyond was demonstrated by [38], in which taste-active compounds in roasted cocoa nibs were identified by means of bioassay-guided fractionation. In this context, sequential application of solvent extraction, gel permeation chromatography, and RP-HPLC in combination with taste dilution analyses [39], followed by LC-MS and 1D/2D-NMR experiments, ultraviolet/visible (UV/Vis), circular dichroism (CD) spectroscopy, and polarimetry, as well as independent enantiopure synthesis revealed a family of previously unidentified amino acid amides. Accurate quantitation of *N*-phenylpropenoyl-L-amino acids was performed by stable isotope dilution analysis (SIDA) using LC-MS/MS. A total of 84 putative taste compounds were quantified in roasted cocoa beans, and then rated for taste contribution on the basis of dose-over-threshold (DoT)-factors in order to bridge the gap between pure structural chemistry and human taste perception. To verify these quantitative results, an aqueous taste reconstitute was prepared by blending aqueous solutions of the individual taste compounds in their “natural” concentrations. Sensory analyses revealed that the taste profile of this artificial cocktail was very close to the taste profile of an aqueous suspension of roasted cocoa nibs. To further narrow down the number of key taste compounds, taste omission experiments and human dose/response functions were performed demonstrating the key organoleptics of the roasted cocoa nibs [38,40,41,42]. Additional bioactivity screening of selected isolated or synthesized *N*-phenylpropenoyl-L-amino acids from cocoa revealed induction of mitochondrial activity and proliferation rate in human liver cell lines as well as human keratinocytes [43], and potent inhibition of the adhesion of *Helicobacter pylori* to human stomach tissue [43,44]. 

Bioactivity-guided separation/isolation of already known bioactive compounds should be avoided. This process is called dereplication. Dereplication strategies generally involve a combination of bioassay, separation science, spectroscopic methods, and database searching and can be regarded as chemical or biological screening processes [45]. The identification of known compounds that could be responsible for the bioactivity of an extract under investigation is the first critical step prior to bioactivity-guided isolation. This can mean either full identification of a compound after only partial purification, or partial identification to the level of a class of compounds. Full identification in these cases relies on comparison with a characterized reference compound. Partial identification serves to: (a) identify undesirable compounds, such as tannins, polyphenols, and fatty acids; (b) prioritize samples for extraction; and (c) gather information on the type of compound to facilitate subsequent isolation [45]. If the dereplication process reveals that the bioactive component of an extract or subfraction is already known and has the same, or similar, bioactivity as the whole extract how does one then proceed? Is this a reason for excluding further bioactivity-guided separation of this extract? How does the activity of your extract compare with that of the single known compound? Even when the same bioassay is used for a certain extract, and a known or exactly this identified bioactive used as control, could you be really sure that this is the only one bioactive constituent in your extract or not? 

To avoid replication the following four points should be considered.

1. It is necessary to know the concentration of the already described bioactive component in the extract that you are investigating. This enables a correlation of the activity of crude extract with the activity of an individual, identified compound.
2. Hundreds, or thousands, of other compounds may also be present in the extract or subfraction(s); it might be unclear if there are suppressive, additive and/or synergistic effects.
3. Most isolated natural products are chrial, giving rise for a number of stereoisomers, which could not (or only partially) be determined via LC-UV, LC-MS and NMR. The bioactivity of enantiomers could be totally different, e.g., thalidomide [46].
4. One needs to know if the undesirable compounds, such as tannins, polyphenols, and fatty acids are present in the extract. For example, polyphenols are known antioxidatives [34,35,47]. By removing the polyphenols in the context of screening for other antioxidants, new polyphenol antioxidants might be excluded. It is known that small changes in the molecular structure, like stereochemistry or constitution isomers, could decisively influence the bioactivity. The diversity of tannins is so high, that the different bioactivities could not be predicted. To summarize, bioactivity-guided fractionation is complex, cumbersome, and expensive but necessary. All the factors that affect de-replication should be analyzed prior to considering the isolation procedure, otherwise there is potential of failure to detect new bioactive compounds.

### 2.5. Observational and Clinical Research

Observational and Clinical Research is fundamental to validate treatment methods, safety, estimate efficacy and doses, and demonstrate and document medicinal effects of crude extracts, fractions, sub-fractions, and isolated compounds prior to using them to improve the health and wellness of veterinary patients in real practice settings. This research assures the removal of toxic and inhibitory components, while leaving those with synergistic or additive beneficial effects allowing the production of refined products with concentrated active constituents in the mixture of extracts or composite formula drugs. This research is needed to shift TAHM towards “evidence-based traditional African medicine” hence, make way for the acceptance of extracts and isolated compounds in medical practices in Africa and integration into western medicine. Chinese traditional medicine has advanced using varying proportions (combinations) of different plant parts/plant species packaged as tablets or capsules [29,48,49]. Standardized, formulated plant extracts for adjunctive treatment with acupuncture and western medicine are needed to treat gastrointestinal illnesses with chronic pain and inflammation in veterinary patients. New products should be formulated using efficacious and proven natural remedies with the goal of reducing hospitalization time, treatment time, and cost.

## 3. Parallels in Traditional Herbal Medicine in Africa and China

### 3.1. Overview

Traditional healing methods are integral components of health care in Africa and China [1,2,3,9,14,48,49,50,51,52,53,54]. Historically, traditional treatments of veterinary patients rely on extracts (90%) of plant origin and there is overlap of plant species used in these practices (Table 1, Table 3 and Table 4). African and Chinese ethnoveterinary practices are not widely accepted in the western world, nor are they fully exploited internally by medical professionals [1,2,3,7,9,14,48,49,50,51]. Veterinary health care professionals need to be pro-active in translational research with the goal of better understanding and higher integration of traditional healing with western practices. The key barriers hampering the integration into western medicine are similar [2,3,7,9,12,48,49,50,51].

### 3.2. What Africa Can Learn from TCM

African countries have much to learn from the milestones made in traditional Chinese medicine and Chinese ethnoveterinary medicine. Several themes emerge in support of this idea: African countries should support and promote basic and clinical research in TAHM to define evidence for efficacy, safety, quality, and mechanisms of action for plant-based traditional therapies. Research needs to be aimed at developing medications for companion and pet animals, which appear to be less documented but much needed in western medicine. Documentation of plant species *versus* diseases conditions, safety, dose toxicity, bioactive compounds and their chemical structures, and mechanisms of action are all essential for product development, academic and prescriptions purposes. This data should be compiled in electronic databases to ease accessibility, and compiling TAHM pharmacopoe should be done at national and continental levels. 

Compound formulas (herbal mixtures) constitute the majority of TCVM prescriptions containing principal herbs, associate herbs, adjuvant, and messenger herbs. Processed formulations have reduced toxicity and concentrated bioactive ingredients [31,48,50]. There are indications that some parts of Africa have begun manufacturing natural extracts and semi-purified formulations [12,28] and this should be a pan African goal. However, the efficacy, safety, quality and advantages of these products should be established, and processing must be adequately regulated [27,28,51]. Traditional Chinese medicine is taught in traditional medical and veterinary schools [27,52]. Incorporating ethnomedicine in traditional veterinary medicine curricula is much needed in Africa. This will increase the awareness of folk medicine amongst veterinary healthcare and animal production professionals, and have a positive impact on future research and development strategies. China has established a TCM regulatory institution to ensure the quality and safety of patients [14,26,29,48] and this must be done in African countries.

**Table 1:** Inventory of African herbal and plant therapies of animal pain and diarrhea.

Species	Family name	Part used	Diseases or symptoms	Reference	Tests: analgesia, inflammation and diarrhea	Compounds/class
*Achyranthes bidentata *Blume	Amaranthaceae *		Abdominal pain and arthritis	[6]		Triterpenoid and saponin
*Celosia trigyna *L*.*^ †^	Amaranthaceae	Whole plant- used in a mixture	Diarrhea, dysentery, pain arthritis and neuralgia	[4]		
*Agapanthus praecox *Willd.	Amaryllidaceae		Small ruminants diarrhea	[53]		
*Ozoroa paniculosa *(Sond.) R. & A. Fernandes	Anacardiaceae *	Bark and roots	Diarrhea	[54]		Anacardic acid, ginkgoic acid and triterpenes
*Protorhus longifolia *(Bernh.) Engl.	Anacardiaceae		Cattle diarrhea	[53]		
*Scleoracarya birrea *(A. Rich.)	Anacardiaceae *	Bark	Diarrhea	[54]	Paw edema, heat-induced pain [55]	Flavonol, epicatechin derivatives and tannins
*Spondias mombin*	Anacardiaceae	Leaves	Diarrhea	[56]		
*Rhus lancea *L.F	Anacardiaceae	Roots/bark	Diarrhea	[54]		Flavonoid and tannins
*Annona senegalensis *Pers*.*	Annonaceae *	Essential oils from leaves	Diarrhea and dysentery, toothaches, and tonic	[53,57,58]	Paw oedema, heat-induced pain [59,60]; anti-motility and anti-cholinergic tests	
*Centella asiatica *(L.) Urb*.*	Apiaceae *	Whole plants used in a mixed formulation	Diarrhea and dysentery	[4]		
*Acokanthera oppositifolia *(Lam.) Codd	Apocynaceae *	Root decoctions	Pain and diarrhea	[54]		Polyphenolics and cardiac glycosides
*Calotropis procera *Aiton. F	Apocynaceae	Leaves	Stomach pain	[61]		
*Landolphia heudelotii *A. DC*.*	Apocynaceae	Leaves, bark	Diarrhea	[61]		
*Saba senegalensis *(A.DC.) Pichon	Apocynaceae	Leaves	Diarrhea	[61]		
*Sarcostemma viminale*	Apocynaceae	Stem	Diarrhea; increase productivity	[62]	Improve livestock productivity (milk) [63]	
*Gomphocarpus fruticosus *(L.) W.T.Aiton	Apocynaceae	Leaves	Diarrhea	[64]		
*Hydrocotyle mannii *Hook.f.	Araliaceae	Leaves in a mixtures of leaves from other species	Diarrhea and dysentery	[4]		
*Aloe marlothii *A.Berger	Asparagaceae *	Leaves	Diarrhea	[65]		
*Cordyline terminalis *var. *cannifolia *(R.Br.) Benth.	Asparagaceae	Stem and root barks used in mixed preparations *Trema orientalis* (L.) Blume	Diarrhea, arthritis, fracture, neuralgia, rheumatism, sprain	[4]		
*Ledebouria revoluta *(L.f.) Jessop	Asparagaceae *	Leaves mixed with leaves from other species; bulb	Diarrhea; ruminant diarrhea	[4,53]		Homoisoflavanones and chalcones
*Brachylaena ilicifolia *(Lam.) Phillips & Schweick	Asteraceae	Leaves	Diarrhea (lambs)	[53]		
*Markhamia tomentosa *(Benth.) K.Schum. ex Engl.	Bignoniaceae *	Roots and leaves	Diarrhea, dysentery, as febrifuge, painful and inflammation	[66]		
*Heliotropium indicum *L*.*	Boraginaceae	Leaves	Acute diarrhea	[61]		
*Cynoglossum coeruleum *Hochst. ex A.DC.	Boraginaceae	Crushed roots of *Rumex nepalensis*, *Carduus nyassanus* and *C. coeruleum* water extract	Diarrhea	[64]		
*Erucastrum arabicum* Fisch. & C.A.Mey. ^†^	Brassicaceae	Whole plant- used in a mixture with other plants	Bloody diarrhea, arthritis, fracture and neuralgia	[4]		
*Lobelia mildbraedii *Engl.	Campanulaceae	Ground leaves mixed with several other species	Diarrhea	[4]		
*Humulus lupulus *L.	Cannabaceae *	Seeds	Diarrhea, pain and sedative	[67,68]	COX-2 inhibition and arthritis in mice [69]	Phenolics and proanthrocyanidins [70]
*Trema orientalis *(L.) Blume ^†^	Cannabaceae	Leaves used in mixed preparations	Diarrhea, arthritis, fracture, and, neuralgia	[4]		
*Capparis tomentosa *Lam.	Capparaceae	Roots	Diarrhea	[53]		3-Hydroxy- 4-methoxy-3-methyl-oxindole [71]
*Maytenus heterophylla *(Eckl. & Zeyh.) N.Robson	Celastraceae	Leaf and bark	Diarrhea	[53]		Dulcitol, a spermidine alkaloid, celacinnine, triterpenoids and maytansine [72,73]
*Maytenus senegalensis *(Lam.) Exell	Celastraceae *	Leaves	Diarrhea	[61]		
*Cassia sieberiana ^€^*	Cesalpiniaceae	Leaves, root, bark	Intestinal colic	[56]		
*Anogeissus leiocarpus*	Combretaceae	Leaves	Abdominal pain	[56]		
*Guiera senegalensis *J. F. Gmel	Combretaceae *	Stem, bark	Diarrhea	[61]		
*Terminalia sericea *	Combretaceae *	Root decoctions	Diarrhea	[54,65]	Indian Arjuna has been tested for pain	Flavonoids and triterpenoids
*Artemisia herb-alba*	Compositae	Aerial parts	Diarrhea			Essential oils
*Berkheya spekeana *Oliv.	Compositae *	Leaves mixed with several other spp	Diarrhea	[4]		
*Bothriocline ugandensis *S. Moore) M.G.Gilbert	Compositae	Ground leaves mixed with several other species	Diarrhea	[4]		
*Brachylaena ilicifolia *(Lam.) Phillips & Schweick.	Compositae *	Leaves	Lamb diarrhea, pain	[53]		
*Crepis rueppellii *Sch.Bip.	Compositae	Leaves	Diarrhea	[64]		
*Melanthera scandens * ^†^	Compositae	Leaves used in a mixed preparation	Diarrhea and dysentery	[4]		
*Schkuhria pinnata *(Lam.) Kuntze ex Thell.	Compositae	Aerial parts	Diarrhea	[53]		
*Senecio mannii *Hook.f.	Compositae	Whole plants- mixed with other plants	Diarrhea, arthritis, fracture and neuralgia	[4]		
*Tagetes minuta *L. ^†^	Compositae	Leaves used in a mixed preparation	Diarrhea and dysentery	[4]		
*Vernonia amygdalina *Delile^†^	Compositae *	Leaves	Diarrhea, dysentery, pain	[52,64]	Writhing, formalin, and tail-flick tests [74]	Polyphenols and sesquiterpene lactones [75]
*Vernonia kirungae *R.E.Fr.^†^	Compositae	Leaves- mixed with stem of *Musa sapientum*	Diarrhea, arthritis, fracture, and neuralgia	[4]		
*Lagenaria abyssinica *(Hook. f.) C. Jeffrey	Cucurbitaceae	Ground leaves mixed with several other species	Diarrhea	[4]		
*Mukia maderaspatana *(L.) M.Roem.	Cucurbitaceae	Ground leaves mixed with several other species	Diarrhea	[4]		
*Juniperus phoenicea *L.	Cupressaceae	Decoction of leaves	Diarrhea	[4]		
*Juniperus procera *Hochst. ex Endl.	Cupressaceae	Leaves	Diarrhea	[64]		
*Cupressus lusitanica*	Cupressaceae	Water extract from leaves of *Vernonia amygdalina*, *Millettia ferruginea* and *Gomphpcarpus fruticosus*; and roots of *Juniperus procera*, *Cupressus lusitanica *and *Crepis rueppellii*.	Diarrhea	[64]		
*Nephrodium filix-mas * ^†^	Dryopteridaceae	Rhizomes of ferns in a mixed preparation	Diarrhea/dysentery	[4]		
*Diospyros mespiliformis *Hochst. Ex ^†^	Ebenaceae	Leaves, unripeFruit, bark	DiarrheaMilk production	[61,76]		
*Jatropha zeyheri *Sond.	Euphorbiaceae	Root decoctions	Diarrhea	[54,76]		Flavonoids, saponins, phorbo esters and triterpenoids
*Ricinus communis *L.^†^	Euphorbiaceae	Oil- mixed with *Trema orientalis (L.) Blume Cordyline terminalis *whole plant mixed with other plants- ashes	Arthritis, fracture, and neuralgia	[4]		
*Pelargonium odoratissimum *(L.) L'Hér.	Geraniaceae		Diarrhea	[53]		Flavonoids tannins, coumarins and phenolic acids
*Pelargonium sidoides *DC. ^†^	Geraniaceae *		Diarrhea in horses	[54]		
*Pelargonium reniforme *Curtis ^†^	Geraniaceae *		Goat and cattle diarrhea, dysentery	[23,54]		Anthrocynins, coumarins, flavonoids, proanthrocynins and diterpene
*Harungana madagascariensis *Lam. ex Poir.	Hypericaceae *	Leaves mixed with other plants	Diarrhea	[4]		
*Hypericum perforatum*	Hypericaceae *	Pods, aerial parts	Analgesic, psychomotor disturbances	[67,70,77]		Phenolics and hyperforin [70]
*Hypericum revolutum *Vahl	Hypericaceae	Leaves mixed with other plants	Diarrhea	[4]		
*Crocosmia paniculata *(Klatt) Goldblatt	Iridaceae	Corm	Bovine diarrhea	[53]		
*Watsonia densiflora *Baker	Iridaceae	Corm	Calf diarrhea	[53]		
*Watsonia tabularis *J.W.Mathews & L.Bolus	Iridaceae	Corm	Calf diarrhea	[78]		
*Clerodendrum myricoides *R. Br. ^†^	Lamiaceae *	Leaves used in a mixed formula with *Sida rhombifolia *L. leaves	Diarrhea and dysentery	[4]		
*Marrabium vulgare*	Lamiaceae	Decoction	Diarrhea			Marrubin, choline, tannins, essential oils and glucosides
*Plectranthus barbatus*	Lamiaceae	Leaves mixed with other plants	Diarrhea	[4]		
*Pycnostachys erici-rosenii*	Lamiaceae	Leaves mixed with other plants	Diarrhea	[4]		
*Rotheca myricoides *(Hochst.) Steane & Mabb.	Lamiaceae	Root, bark	Cattle diarrhea	[79]		
*Ocimum lamiifolium *Hochst. ex Benth.	Lamiaceae	Water extract of fresh leaves of *Vernonia amygdalina* and *Clutia abyssinica*; and root of *Ocimum lamifolium*	Diarrhea	[64]		
*Tetradenia riparia *(Hochst.) Codd^†^	Lamiaceae	Whole plant or dried flowers-mixed with other plants	Diarrhea, aArthritis, fracture and neuralgia	[4]		
*Acasia ataxacantha *DC.	Leguminosae	Bark/decoction	Diarrhea	[56]		
*Acacia karroo *Hayne ^†^	Leguminosae	Bark and leaves	Diarrhea (poultry, ruminants and pigs)	[53,65]		
*Acacia nilotica *(L.) Willd. Ex	Leguminosae	Leaves	Diarrhea	[61]		
*Acacia polyacantha*	Leguminosae *	Root	Body pain	[52,80]		
*Acasia senegal *(L.) Willd.	Leguminosae	Rubber/ latex	Intestinal pain	[56]		
*Acasia raddiana; Acacia tortilis subsp. raddiana *(Savi) Brenan	Leguminosae	Leaves	Diarrhea-camels			
*Calpurnia aurea * ^†^	Leguminosae *		Cattle diarrhea, dysentery	[54]		Quinolizidine
*Cassia occidentalis *L	Leguminosae *		Body pain (tonic ruminants)	[61]		
*Cassia siamea *Lam.	Leguminosae	Leaves	Stomach pains	[61]		
*Ceratonia siliqua*	Leguminosae		Diarrhea			
*Dichrostachys glomerata*	Leguminosae *	Leaf	Diarrhea and pain	[52]		Alkaloids, phenols and tannins
*Elephantorrhiza burkei *Benth. ^†^; *Elephantorrhiza elephantina *(Burch.) Skeels ^†^	Leguminosae *	Aerial parts, roots and bulb	Abdominal pain/ diarrhea, dysentery, (horse and ruminants)	[53]		
*Indigofera spp*	Leguminosae *	Whole plants/ roots	Gastrointestinal pain	[53]		
*Kotschya africana* ^†^	Leguminosae	Whole plant- mixed with other plants	Gastrointestinal pain, arthritis, fracture, neuralgia, rheumatism, sprain	[4]		
*Lonchocarpus laxiflorus*	Leguminosae	Bark/decoction	Diarrhea	[56]		
*Millettia ferruginea *(Hochst.) Bak.	Leguminosae	Leaves	Diarrhea	[64]		
*Parkia biglobosa ^€^*	Leguminosae *	Seeds, fruits, roots, bark/decoction	Pain and diarrhea	[56]	Writhing test- effective/ not effective in hot-plate [81]	
*Phaseolus vulgaris *L.	Leguminosae	Fruits	Diarrhea	[4]		
*Pterocarpus erinaceus *Poir.	Leguminosae *	Bark of tillage	Diarrhea	[56]	Edema + writhing tests [82]; diarrhea, charcoal meal transit time [83]	
*Peltophorum africanum *Sond. ^†^	Leguminosae *	Root and stem bark	Cattle diarrhea, dysentery, colic and pain	[54,84]		Flavonoids, coumarins, tannins gallic and chlorogenic acid, Flavonol glycosides and flavonol glucoside gallates
*Pterocarpus erinaceus *Poir.^†^	Leguminosae *	Leaves, roots, bark	Diarrhea	[56]		
*Senna italica *Mill.	Leguminosae *	Stem bark	Cattle diarrhea,	[54,76]		
*Taverniera abyssinica *A.Rich.	Leguminosae *	Root	Pain	[64]	Hot plate, writhing [85]	Phytoalexins and isoflavonoids
*Tephrosia vogelii *Hook.f. ^†^	Leguminosae	Whole plants used in a mixed preparation	Diarrhea, dysentery	[4]		
*Xeroderris stuhlmannii *(Taub.) Mendonça & E.P. Sousa	Leguminosae	Bark	Abdominal pain	[56]		
*Strychnos henningsii *Gilg	Loganiaceae *	Bark	Cattle diarrhea	[53]		
*Tapinanthus bangwensis *(Engl. & K.Krause) Danser	Loranthaceae *	Leaves	Stomach pain	[52]		
*Lawsonia alba*	Lythraceae		Diarrhea			Xanthones, triterpenoids and napthoquinones
*Adansonia digitata *L. ^†^	Malvaceae *	Leaf/bark/fruit	Diarrhea, stomach pain: diarrhea (fruit)	[52,56]	Hot plate; human trial against diarrhea [86]	
*Sida alba *Forrsk ^†^	Malvacea *	Leaves	Diarrhea, dysentery, pain	[57,58]		
*Sida rhombifolia *L. ^†^	Malvacea	Leaves used in a mixed formula with *Clerodendrum myricoides R. Br.* Leaves	Diarrhea and dysentery	[4]		
*Urena lobata *L.	Malvacea	Leaves used in a mixed preparation	Diarrhea	[4]		
*Khaya senegalensis *(Desv.) A.Juss.^†^	Meliaceae	Stem, bark	Abdominal pain, diarrhea and dysentery; feed supplements	[56,61]		
* Cissampelos mucronata *A.Rich. ^†^	Menispermaceae *	Whole plant- mixed with other plants, roots	Arthritis, fracture, neuralgia, rheumatism, sprain, pain, sedative	[4,52,87]		Alkaloids, sterols, triterpenes, tannins, carbohydrates, glycosides and flavonoids
*Cissampelos torulosa *E.Mey. ex Harv. & Sond. ^†^	Menispermaceae	Leaves	Diarrhea, dysentery	[57,58]	*Cissampelos pareira* leaves tested for anti- depressant effects in mice and rats	
*Ficus thonningii *Blume	Menispermaceae *	Crushed leaves mixed with stem barks of *Myrica kandtiana*	Diarrhea	[4]		
*Ficus exasperata *Vahl	Moraceae	Crushed leaves mixed with stem barks of *Myrica kandtiana*	Diarrhea	[4]		
*Ensete ventricosum *(Welw.) Cheesman	Musaceae	Leaves mixed with other plant leaves	Diarrhea	[4]		
*Musa sapientum *L. ^†^	Musaceae	Stem mixed with leaves of *Vernonia kirungae* R.E.Fr.	Arthritis, fracture, neuralgia	[4]		
*Myrica kandtiana*	Myricaceae	Leaves, stem and bark used with *Ficus spp* in mixed preparations	Diarrhea	[4]		
*Psidium guajava *L. ^†^	Myrtaceae *	Leaves in a mixed formulation, leaves	Diarrhea, dysentery	[4,88]		
*Syzygium guineense*	Myrtaceae *	Leaf, stem bark, root	Pain, sedation	[88]		
*Syzygium cordatum*	Myrtaceae	Bark and leaves	Diarrhea	[57,58]		
*Nymphaea calliantha *Conard. ^†^	Nymphaeaceae	Leaves in a mixed preparation	Bloody stool and diarrhea.	[4]		
*Ximenia caffra *Sond ^†^	Olacaceae *	Leaves and root	Diarrhea and dysentery	[57,58]		
*Striga hermonthica *(Delile) Benth.	Orobanchaceae	Whole plant/decoction	Diarrhea	[56]		
*Bridelia micrantha *Baill ^†^	Phyllanthaceae *	Root, bark and seeds	Pain, arthritis and diarrhea,	[57,58]	Castor-oil diarrhea, charcol meal anti-motility tests in rats [89]	
*Bridelia micrantha *(Hochst.) Baill. ^†^	Phyllanthaceae *	Leaves in a mixed formulation	Diarrhea, dysentery	[4]		
*Clutia spp, Clutia pulchella *L.	Peraceae *	Leaves	Painful joints	[84]		
*Piper capense *L.f.	Piperaceae	Milk infusions ofleaves, stems and roots	Diarrhea, pain in calves	[4]		
*Pittosporum viridiflorum*	Pittosporaceae	Stem, bark	Pain	[84]		Saponins and sesquiterpenoids
*Plumbago auriculata *Lam.	Plumbaginaceae *	Roots	Cattle diarrhea	[53]		Naphthoquinone and plumbagin [84]
*Coix lacryma-jobi *L. ^†^	Poaceae	Roots in a mixed preparation	Diarrhea, dysentery	[4]		
*Protea caffra *Meisn.	Poaceae	Root, bark (enema)	Bloody diarrhea in calves	[53,54]		
*Protea welwitschii *Engl. ^†^	Poaceae *	Root, bark	Dysentery, diarrhea in calves	[53]		
*Sorghum bicolor *(L.) Moench	Poaceae *	Powder added to plant leaves mixture; germed seeds	Diarrhea, dysentery	[4,56]		
*Rhynchelytrum repens *(Willd.) C.E.Hubb. ^†^	Poaceae	Leaves used in mixed formula	Dysentery and diarrhea	[4]		
*Podocarpus falcatus *(Thunb.) R. Br. ex Mirb.^†^	Podocarpaceae	Leaf	Canine distemper diarrhea	[24,53]		
*Ramalina farinacea *(L.) Ach.	Ramalinaceae		Analgesic, anti-inflammatory	[56]		
*Ziziphus zeyheriana *Sond.	Rhamnaceae	Root	Diarrhea	[65]		
*Prunus persica *(L.) Stokes	Rosaceae	Root	Diarrhea in small ruminants	[24,53]		
*Cinchona ledgeriana *(Howard) Bern.Moens ex Trimen ^†^	Rubiaceae	Leaves mixed with other plant leaves; stem, barks used in a mixed decoction	Diarrhea, dysentery and diarrhea	[4]		
*Sarcocephalus latifolius *(Sm.) Bruce latifolius	Rubiaceae	Roots/decoction	Diarrhea	[56]		
*Ptaeroxylon obliquum *(Thunb.) Radlk.	Rutaceae *	Leaves, stem bark and root	Diarrhea, dysentery, arthritis and pain	[84]		Essential oil, resin, saponin, pyrogallol, tannins flavone and alkaloids, coumarins
*Hippobromus pauciflorus *Radlk. ^†^	Sapindaceae *	Bark, root and leaves	Diarrhea, dysentery, analgesic	[5,24,53]		
*Vitellaria paradoxa *C. F. Gaertn. ^†^	Sapotaceae	Leaves	Bloody diarrhea	[61]		
*Nicotiana tabacum *L.	Solanaceae	Leaves mixed with other plant leaves	Diarrhea	[4]		
*Solanum panduriforme *E. Mey.	Solanaceae *	Leaf infusions, fruit sap	Diarrhea	[58,65]		
*Withania somnifera *(L.) Dunal	Solanaceae *	Roots	Diarrhea	[65]		Steroids; witherferin, choline, tropanaol, glycowithanolides, withanolides, withaferine and withasomnine [70,72]
*Waltheria indica *L.	Sterculiaceae	Leaves	Diarrhea, tonic	[61]		
*Urtica doica *L.	Urticaceae	Stem and leaves	Diarrhea, pain, rheumatism, inflammation			
*Pouzolzia mixta *Solms	Urticaceae *	Root, stem and leaves	Diarrhea, dysentery	[57,58]		
*Lippia javanica *(Burm. f.) Spreng. (E.A.) ^†^	Verbenaceae *	Leaves	Dysentery and diarrhea	[57,58]		Pentacyclic triterpenoids, essential oil, amino acids, stearic and other acids
*Rhoicissus tridentata *(L. f.)Wild & R.B. Drumm.	Vitaceae	Root, tubers and fruits	Ruminant diarrhea	[24,53,54]		Irioids, stilbenes, flavonoids and triterpenoids
*Balanites maughamaii*	Zygophyllaceae	Leaves	Cattle diarrhea	[54,76]		

Blanks: Unknown or the existence of this information was not determined through literature search. However, for the majority the listed plant species the information is not known. ^†^ The extracts of these plant species are recommended for high throughput screening for bioactive agents to treat gastrointestinal (GI) disorders such as diarrhea, inflammation and chronic pain. These plants have potential for novel complementary drugs against GI disorders presenting with diarrhea, dysentery, and chronic pain and hence complementary usage with acupuncture and western medications. * Plant species under this family (same row) are used both in human (see Table 2) and veterinary care for GI ailments and pain management.

**Table 2:** An inventory of African herbal therapies against human gastrointestinal pain and diarrhea.

Species	Family name	Part used	Diseases or symptoms	Reference	Analgesia/anti-inflammatory/anti-diarrhea tests	Compounds/class
*Carpobrotus edulis *(L.); *Carpobrotus acinaciformis *(L.)*L. bolus and Carpobrotus muirii (L.) L. bolus *^†^	Aizoaceae	Leaves	Diarrhea and dysentery	[ 5,90]		
*Achyranthes aspera*	Amaranthaceae	Whole plant (root, leaves and aerial parts)	Chest pain and stomach complaints	[ 84]		Achyranthine and glycosides
*Hermbstaedtia odorata*	Amaranthaceae	Leaves	Diarrhea	[ 5]		
*Guilleminea densa *(Willd. ex Schult.) Moq.	Amaranthaceae	Root	Diarrhea	[ 91]		
*Scadoxus puniceus *(L.) Friis & Nordal	Amaryllidaceae	Bulb & roots	Stomach ailments and diarrhea	[ 5]		
*Tulbaghia alliacea *L.f.	Amaryllidaceae	Bulb	Stomach ailments and rheumatism	[ 5]		
*Anacardium* *Occidentale*	Anacardiaceae	Fruit/Bark	Pain and diarrhea	[ 52]	Tested for diarrhea [92]	
*Rhus chirindensis * ^†^	Anacardiaceae	Stem bark	Stomach ailments, and diarrhea; inflammation, rheumatism, analgesic and neurologic complaints	[ 84]	Hot-plate and acetic acid-induced pain and egg albumin-induced pedal edema [93]	Flavonoids, triterpenoids
*Mangifera indica *L*.*^ †^	Anacardiaceae	Stem bark	Diarrhea and dysentery; inflammation and neuropathic pain	[ 52,94]	Tail flick, writhing tests carrageenan- and formalin-induced oedema [95,96]	Polyphenols
*Protorhus longifolia * *(Bernh. ex C. Krauss) Engl.*	Anacardiaceae	Bark	Diarrhea and dysentery	[ 5]		
*Sclerocarya birrea* (A. Rich.) Hochst ^†^	Anacardiaceae	Root, leaves and stem bark	Diarrhea, dysentery and pain		Egg albumin-induced paw oedema and heat-induced pain [55,62]	Tannins, alkaloids, vitamin C and flavonoids
*Lannea schimperi (*Hochst. ex A.Rich.) Engl.	Anacardiaceae	Roots/bark leaves; Bark	Stomach ache, chronic diarrhea	[ 94,97]		
*Ozoroa insignis *Delile	Anacardiaceae	Roots/stem bark/leaves	Diarrhea and stomach ache	[ 91,94]		
*Ozoroa paniculosa *(Sond.) R. Fern. & A. Fern.	Anacardiaceae	Bark/root bark	Diarrhea and abdominal pain	[ 53,65]		
*Searsia incica, *L.F*. Searsia leptodictya (Diels*) and other related species*.*	Anacardiaceae	Root/bark/leaves	Diarrhea and pain	[ 53,57,65]		Flavonoids
*Annona senegalensis * ^†^	Annonaceae	Leaves	Diarrhea (bark), toothaches and body pain	[ 52]	Egg albumin-induced paw oedema heat-induced pain [59,60] (antimotility mice; anti-cholinergic in rabbits)	
*Annona senegalensis *Pers.	Annonaceae	Roots	Stomachache	[ 94]		
*Uvaria chamae *P.Beauv. ^†^	Annonaceae	Roots/stem bark	Gastroenteritis, diarrhea, dysentery, abdominal pain; sedative and analgesic	[ 52,98]	Paw edema [98]	Alkaloids, flavonoids, tannins, saponins and phenols [98]
*Foeniculum vulgare* Mill. ^†^	Apiaceae	Stem/leaves	Cramp; colic, diarrhea	[ 84]	[99]	Quercetin derivatives and volatile oils
*Heteromorpha trifoliate* (H.L.Wendl.) Eckl. & Zeyh ^†^	Apiaceae	Root/leaves	Diarrhea; anti-inflammatory; painful joints, backache and headache	[ 84,100]	COX-1 inhibition test: [101]; TPA-induced ear and carrageenan-induced paw oedema in mouse	Falcarindiol and sarisan
*Alepidea amatymbica* Eckl. & Zeyh.^†^	Apiaceae	Root/rhizome	Diarrhea, headache and rheumatism	[ 84,102]	[102]	Terpenoids
*Centella spp*	Apiaceae	Roots	Diarrhea and dysentery	[ 23,28]		
*Calotropis procera*	Apocynaceae	Leaves	Diarrhea and pain	[ 52]		Alkaloids, cardiac glycosides, tannins, flavonoids, sterols and/or triterpenes in aerial parts [103]
*Cynanchum acutum *L. ^†^	Apocynaceae	Aerial parts	Diarrhea	[ 104]	Castor oil- induced diarrhea; anti-motility assay	Tannins, flavonoids, unsaturated sterols, triterpenes, carbohydrates, lactones and proteins and amino acids
*Landolphia heudelotii *A.DC.	Apocynaceae	Roots	Body pain and diarrhea	[ 52]		
*Acokanthera oppositifolia* (Lam.) Codd	Apocynaceae	Roots/leavs	Stomach ache, diarrhea, painful feet, rheumatism andtoothache	[ 5,84]		Amorphous acokantherin, flavonoids and proanthocyanidins [105]
*Asclepias fruticosa*	Apocynaceae	Roots/stem/leaves	Stomach pain and diarrhea	[ 84]		Aardenolide glycoside, steroidal glycosides and 5,11-epoxymegastigmanes [106]
*Amorphophallus consimilis *Blume	Araceae	Bark	Diarrhea	[ 52]		
*Xysmalobium undulatum *(L.)^†^	Apocynaceae.	Roots	Diarrhea, dysentery, stomach pain and headaches	[ 5]		
*Aloe ferox*	Asparagaceae	Leaves	Diarrhea	[ 5,107]		
*Aloe greatheadii *Schönland	Asparagaceae	Leaves	Diarrhea,	[ 107]		
*Eucomis autumnalis *(Mill.) Chitt. ^†^	Asparagaceae	Bulb	Abdominal pain and diarrhea; and back pain	[ 5]		
*Eucomis comosa* ^†^	Asparagaceae	Root/bulb	Rheumatism and teething baby	[ 84]		Homoisoflavones, nortriterpenes, and eucosterol
*Ledebouria ovatifolia*	Asparagaceae.	Bulb	Gastroenteritis and backache	[ 84]		Bufadienolides
*Scilla nervosa * ^†^	Asparagaceae.	Bulb	Dysentery and rheumatism	[ 84]		Digitalis, homoisoflavonoids and stilbenoids [108]
*Kigelia Africana * ^†^	Bignoniaceae	Bark/dried fruit	Diarrhea; painful joints, back and rheumatism	[ 84]	Castor oil-induced diarrhea Antimotility [109]; Writhing and paw edema tests [110]	Luteolin, flavonoids isocoumarins, sterols and iridoid glycosides, saponins, carbohydrates, glycosides and reducing sugars
*Markhamia tomentosa *(Benth.) K.Schum. ex Engl.	Bignoniaceae	Leaves	Diarrhea	[ 66]		
*Sarcophyte sanguinea * ^†^	Balanophoraceae	Whole plant	Diarrhea, dysentery and pain	[ 84]		Exocarpic acid, naringenin,
*Tecomaria capensis * ^†^	Bignoniaceae	Bark/leaves	Diarrhea, dysentery, stomach pains; and chest pains	[ 84]		Flavonols, alkaloids and tannins
*Diplotaxis acris *(Forssk.) Boiss. ^†^	Brassicaceae	Aerial parts	Diarrhea	[ 104]	Castor oil induced diarrhea	Tannins, flavonoids, unsaturated sterols, triterpenes, carbohydrates, lactones and proteins/amino acids
*Schouwia thebaica * ^†^	Brassicaceae	Aerial parts	Diarrhea	[ 104]	Castor oil induced diarrhea, anti-motility assay	Tannins, flavonoids, unsaturated sterols, triterpenes, carbohydrates, lactones and proteins/amino acids
*Boscia salicifolia *Oliv.^†^	Capparaceae	Roots/bark	Diarrhea, pain, rheumatism	[ 94]		
*Capparis erythrocarpos *Isert ^†^	Capparaceae	Roots	Chronic diarrhea	[ 97]		
*Humulus lupulus *L. ^†^	Cannabaceae	Seeds	Diarrhea, inflammatation, pain, sedative	[ 68,70]	COX-2 inhibition and arthritis in mice [69]	Phenolics and proanthrocyanidins [70]
*Catha edulis* *’ * ^†^	Celastraceae	Bark/leaves	Pain and amoebic dysentery	[ 64]	Hot-plate, tail-flick, and writhing tests in mice [111]	
*Maytenus senegalensis *(Lam.) Exell	Celastraceae	Roots/bark	Stomach ache	[ 94]		
*Gymnosporia senegalensis (Lam.) Loes.*	Celastraceae		Diarrhea	[ 62]		
*Allanblackia gabonensis (Pellegr.) Bamps * ^†^	Clusiaceae	Stem bark	Diarrhea, dysentery; inflammations and pain		Hot-plate, tail-flick, writhing, paw edema tests [112]	
*Allanblackia floribunda var. gabonensis Pellegr. * ^†^	Clusiaceae	Stem bark/fruit	Dysentery, diarrhea, toothache	[ 113]		
*Garcinia buchananii Baker * ^†^	Clusiaceae	Stem bark	Diarrhea, dysentery, abdominal pain	[ 37,114]	Inhibits GI motility and neurotransmission, lactose diarrhea [37,114,115,116]	Biflavanones, alkaloids and steroids [34,37]
*Garcinia livingstnei *T.Anderson	Clusiaceae	Leaves	Diarrhea	[ 62]		
*Combretum micranthum*	Combretaceae	Leaves	Diarrhea, chest pain	[ 52]		Saponins, glucosides and triterpenes
*Combretum hypopilinum *(Diels)^†^	Combretaceae	Leaves	Bloody diarrhea	[ 52]		Saponins, glucosides and triterpenes
*Combretum zeyheri *Sond. ^†^	Combretaceae	Root	Bloody diarrhea	[ 117]		
*Combretum nigricans*	Combretaceae	Root/leaves	Body pain	[ 52]		Flavonoid, Saponins, glucosides and triterpenes
*Combretum paniculatum /* *C. molle *R. Br. ex G. Don ^†^	Combretaceae	Root/leaves	Diarrhea and pain	[ 52]	Thermally- and chemically-induced nociceptive pain in mice [118]	Saponins, glucosides, triterpenes, flavonoids in similar species inhibit pain [119]
*Terminalia albida *Scott-Elliot ^†^	Combretaceae	Root/leaves	Stomach and back pain	[ 52]	Thermal - induced pain in rat* (Terminalia bellirica* fruits extract)	Furanoid and diterpene
*Terminalia phanerophlebia*	Combretaceae	Root bark	Diarrhea and colic	[ 84]		Triterpenoids, tannin, nerifolin and sericoside
*Terminalia sericae *DC.^†^	Combretaceae	Roots/bark leaves Root bark	Diarrhea and colic, diarrhea, stomachache, limb pain	[ 84,94,120]	Indian Arjuna has been tested for pain	Triterpenoids, tannin, nerifolin and sericoside
*Guiera Senegalensis *J.F.Gmel.^†^	Combretaceae	Roots/leaves	Diarrhea; stomach pain and body pain	[ 52]	Castor oil-induced diarrhea [121]	Alkaloids, steroids and cardiac glycosides [122]
*Combretum zeyheri *Sond.^†^	Combretaceae	Roots/bark/leaves	Diarrhea, dysentery Stomach ache and body pain	[ 94]		
*Acanthospermum australe *(Loefl.)*Kuntze*	Compositae	Whole plant	Diarrhea	[ 62]		
*Artemisia afra Jacq. ex Willd. * ^†^	Compositae		Stomach ailments, and wounds	[ 123]		
*Bidens bipinnata *L. ^†^	Compositae	Leaves, aerial parts	Diarrhea	[ 5,104]	Castor oil diarrhea, anti-motility assay [104]	Tannins, flavonoids, unsaturated sterols, triterpenes, carbohydrates, lactones and proteins/amino acids
*Dicoma capensis *Less.	Compositae	Roots/herbs	Diarrhea	[ 28]		
*Senecio speciosus *Willd.	Compositae	Stem/leaves	Chest pain and headache	[ 84]		
*Vernonia adoensis * ^†^	Compositae	Roots/stem/leaves	Diarrhea and stomach, painful joints, back and chest pain	[ 84,97]		Glaucolides
*Helichrysum spp*	Compositae	Root	Diarrhea	[ 124]		
*Pentzia incana *(Thunb.) Kuntze	Compositae	Root	Diarrhea	[ 28]		
*Printzia pyrifolia*	Compositae	Root	Somach ache	[ 84]		Coumarate
*Berkheya speciosa *(DC.) O.Hoffm.	Compositae	Root/leaves	Abdominal pains	[ 84]		Sesquiterpenoids and socomene
*Bidens pilosa *L. ^†^	Compositae	Root/leaves	Diarrhea, stomach pain, colic	[ 84,125]		Chalchones and polyacetylenes
*Brachylaena elliptica *(Thunb.) Less.	Compositae	Leaves	Stomach and back pain	[ 84]		Mucilage, tannins and onopordopicrin
*Brachylaena discolor *var*. transvaalensis *(E.Phillips & Schweick.) Beentje	Compositae	Bark/leaves	Diarrhea	[ 62]		
*Vernonia amygdalina *Delile^†^	Compositae	Leaves, fruit	Dysentery and pain, chronic diarrhea	[ 52,64,97]	Writhing, formalin test, and tail-flick test [74]	Polyphenols; sesquiterpene and lactones [75]
*Xanthium strumariumv * ^†^	Compositae	Root	Analgesic, dysentery, inflammation	[ 117]	Tested Analgesic [126]	
*Convolvulus fatmensis G. Kunze.* ^†^	Convolvulaceae	Aerial parts	Diarrhea	[ 104]	Castor oil-induced diarrhea; anti-motility assay; anti-nociceptive tests [104]	Tannins, flavonoids, unsaturated sterols, triterpenes, carbohydrates, lactones and proteins/amino acids
*Diospyros mespiliformis Hochst. ex A.DC.*	Ebenaceae	Leaves/unripe fruits	Diarrhea	[ 84]		
*Curtisia dentata *(Burm.f.) C.A.Sm.	Curtisiaceae	Root/bark	Diarrhea and stomach ailments	[ 5]		
*Crassula ovata *(Mill.) Druce/Crassula* tetragona L.*	Crassulaceae.	Leaves	Diarrhea	[ 28]		
*Parinari curatellifolia Planch. ex Benth.* ^†^	Chrysobalanaceae	Root/bark	Chronic diarrhea	[ 97]		
*Diospyros villosa*	Ebenaceae	Roots/leaves	Painful and intestinal complaints	[ 84]		Flavonoids
*Croton gratissimus *^†^	Euphorbiaceae	Stem bark	Diarrhea, dysentery and pain	[ 84,127]		
*Euphorbia h*irta L ^†^	Euphorbiaceae	Leaves	Diarrhea; dysentery	[ 66,128]		Tannins, polyphenols, flavonoids, Alkanes, phytosterols and triterpenes [128]
*Euphorbia cooperi *N.E.Br. ex A.Berger	Euphorbiaceae	Bark of root	Diarrhea and stomach disorders	[ 5]		
*Euphorbia paralias *L.	Euphorbiaceae	Aerial parts	Diarrhea	[ 104]	Castor oil diarhhea, anti-motility assay [104]	Tannins, flavonoids, unsaturated sterols, triterpenes, carbohydrates, lactones and proteins/amino acids
*Pelargonium sidoides *DC.^†^	Geraniaceae	Roots/leaves	Diarrhea, dysentery and vomiting	[ 5]		
*Pelargonium luridum * ^†^	Geraniaceae	Root/leaves	Dysentery	[ 5]		
*Pelargonium reinforme curtis * ^†^	Geraniaceae	Root tuber	Diarrhea, dysentery	[ 28]		
*Pelargonium triste *L'Hér.^†^	Geraniaceae	Tuber	Diarrhea, dysentery	[ 28]		
*Monsonia emarginata *L'Hér.*/Monsonia burkeana *Planch. Ex. Hrv.^†^	Geraniaceae	Roots/herb	Diarrhea dysentery, inflammation	[ 28]		
*Gunnera perpensa *L.	Gunneraceae	Root	Diarrhea, pain	[ 84]	Hot Plate, writhing, and paw edema [129]	Bitter principles
*Hymenocardia acida Tul.*	Hymenocardiaceae	Roots/leaves	Stomach ache	[ 94]		
*Hypericum perforatum*	Hypericaceae	Pods/aerial parts	Diarrhea analgesic, and psychomotor disturbances	[ 70]	Castor oil-induced diarrhea in mice [77]	Phenolic compounds and hyperforin [67,70]
*Harungana madagascariensis *Lam. ex Poir.	Hypericaceae	Bark/leaves	Chronic diarrhea	[ 97]		
*Hydnora africana *Thunb.	Hydnoraceae	Tuber/fruits/leaves	Dysentery and diarrhea	[ 5]		
*Hypoxis latifolia *Hook. (African potato (Eng.) ^†^	Hypoxidaceae	Tuber	Diarrhea and headaches	[ 5]		
*Hypoxis hemerocallidea *Fisch*., *C.A.Mey. & Avé-Lall.	Hypoxidaceae	Tuber	Diarrhea	[ 84]		
*Icacina senegalensis*	Icacinaceae	Leaves	Diarrhea and back pain	[ 52]		Turpentine
*Gladiolus sericeovillosus subsp. calvatus *(Baker) Goldblatt^†^	Iridaceae	Corm	Diarrhea, dysentery and pain	[ 5]		
*Gladiolus dalenii *Van Geel ^†^	Iridaceae	Corm	Diarrhea, dysentery, pain	[ 78]		
*Vitex doniana*	Lamiaceae	Root/leaves	Diarrhea		Castor oil-induced diarrhea [77]	
*Vitex mombassae *Vatke ^†^	Lamiaceae	Root/leaves	Stomach ache and diarrhea	[ 94]		
*Premna senensis *Klotzsch	Lamiaceae	Root/leaves	Stomach ache and body pains	[ 94]		
*Clerodendrum glabrum *E.Mey^†^	Lamiaceae	Root/leaves	Diarrhea; fracture, painful joints and rheumatism	[ 84]	Electric current anxious stimulus [130]	
*Leonotis leonurus*	Lamiaceae	Stem bark/leaves	Dysentery and headache	[ 84]	Heat, acetic acid, egg-edema [95]	Phenolic compounds, resins and carotenoid
*Ocimum gratissimum *L.	Lamiaceae	Leaves	Diarrhea	[ 97]		
*Salvia africana-caerulea *L.	Lamiaceae	Leaves	Diarrhea	[ 28]		
*Ocotea bullata* ^†^	Lauraceae	Bark	Diarrhea; pains and headache	[ 79,84]		Tannins
*Acacia albida*	Leguminosae	Root	Stomach pain	[ 52]		
*Acacia catechu*	Leguminosae	Leaf	Stomach pain	[ 64]		
*Acacia burkei Benth.*	Leguminosae	Root/stem bark	Diarrhea and painful back	[ 84]		
*Acacia mearnsii* De Wild. ^†^	Leguminosae	Bark of root	Diarrhea and stomach disorders	[ 5]		
*Acacia mellifera (M.Vahl) Benth.*	Leguminosae.	Roots/bark/leaves	Stomach ache and diarrhea	[ 96]		
*Acacia polyacantha*	Leguminosae	Root	Body pain	[ 52,80]		
*Acacia sieberiana*	Leguminosae	Stem bark	Diarrhea; and back pains and aches	[ 84]		
*Abrus precatorius L.*	Leguminosae	Root	Stomach ache	[ 94]		
*Albizia harveyi E. Fourn.*	Leguminosae	Roots/leaves	Stomach ache and chest pain	[ 94]		
*Cassia abbreviata Oliv * ^†^	Leguminosae	Roots/bark/leaves	Diarrhea and stomach ache	[ 94]		
*Cassia occidentalis L*	Leguminosae	Leaves	Body pain	[ 52]		
*Calpurnia aurea * ^†^	Leguminosae	Root/bark	Stomach ache and dysentery	[ 64,131]		
*Cassia sieberiana DC * ^†^	Leguminosae	Root/bark	Diarrhea, stomach pains, dewormer, inflammation	[ 52]	Writhing test in mice; and paw oedema in rats [132]	
*Dalbergia nitidula *Baker	Leguminosae	Roots/bark	Diarrhea and toothache	[ 94]		
*Dichrostachys cinerea *(L.) Wight & Arn; .*Dichrostachys cinerea *^†^	Leguminosae	Roots/bark/leaves	Abdominal pains, diarrhea	[ 84]	Writhing tests and castor oil- induced diarrhea [133]	Triterpenoids, beta-amyrim, beta sitosterol, alkaloids and saponin
*Dichrostachys glomerata*	Leguminosae	Leaves	Diarrhea, toothache	[ 52]		Alkaloids, phenols and tannins
*Eriosema psoraleoides *(Lam.) G.Don	Leguminosae	Leaves	Diarrhea, chronic diarrhea	[ 66,97]		
*Erythrophleum lasianthum*	Leguminosae	Stem bark	Body pain and intestinal spasm	[ 84]		Erythrophleine, alkaloids and glucopyranosides
*Elephantorrhiza elephantina *(Burch.) Skeels ^†^	Leguminosae	Root/stem (Mixed with *Acokanthera Oblongifolia)*	Dysentery, diarrhea	[ 5]		
*Indigofera spp*	Leguminosae	Whole plants/roots	Diarrhea	[ 53,91]		
*Moghania faginea (Guill. & Perr.) Kuntze * ^†^	Leguminosae	Root/leaves	Stomach/body/chest pelvic/back pain	[ 52]		Flavonol glycosides
*Mundulea sericea (Willd.) A.Chev.*	Leguminosae	Roots/bark	Stomach ache	[ 94]		
*Parkia biglobosa (Jacq.) * *G.Don * ^†^	Leguminosae	Bark	Diarrhea and tooth ache	[ 52]	Castor oil diarrhea [134] writhing + hot plate tests [81]	Cardiac glycosides, steroids, tannins and alkaloids
*Peltophorum africanum *Sond.	Leguminosae	Root/stem bark	Colic, painful joints, toothaches and backaches	[ 53,84]		Flavonoids, gallic and chlorogenic acid
*Pterocarpus erinaceus *Poir. ^†^	Leguminosae	Leaves	Diarrhea, and body pain	[ 52]	Edema + writhing tests, castor oil-induced diarrhea, intestinal transit time [82,83]	Friedelin, lupeol and epicathechin
*Piliostigma thonningii *(Schum.)^†^	Leguminosae	Leaves/fruit/seed pod	Stomach pain; headache, back and chest pain.	[ 52]		Piliostigmin, 2-phenoxychromone, and C-methylflavonols
*Rhynchosia adenodes *Eckl. & Zeyh.^†^	Leguminosae	Roots	Dysentery and pain	[ 135]		
*Senna italica *Mill.	Leguminosae	Roots/stem bark	Diarrhea	[ 53]		
*Senna occidentalis *(L) Link.	Leguminosae	Roots/leaves	Diarrhea, chronic diarrhea	[ 62,97]		
*Schotia latifolia Jacq./Schotia brachypetala *Sond.^†^	Leguminosae	Bark	Diarrhea and dysentery	[ 62,102,136]		
*Sutherlandia frutescens *L.	Leguminosae	Root, stem bark, leaves/flower	Diarrhea and pain	[ 84]	Hot plate, acetic acid, paw edema tests [55]	Canavanine and free amino-acids, flavonoids and triterpenoids [70]
*Stylosanthes mucronata *Willd.	Leguminosae	Leaves	Diarrhea and chest pain	[ 52]		
*Taverniera abyssinica *A.Rich. ^†^	Leguminosae	Root	Stomach pain and headaches.	[ 64]	Hot plate, writhing [85]	Phytoalexins and isoflavonoids
*Trigonella foenum-graecum *L*.*	Leguminosae	Fruit/leaves	Stomach pain and headaches	[ 64]	Hot plate, writhing, paw edema tests [137]	Flavonol glycosides, alkaloids, cardiac glycosides and phenols
*Zornia milneana* Mohlenbr*. *^†^	Leguminosae	Root/stem/leaves	Diarrhea, dysentery and pain	[ 58]		
*Linum thunbergii* Eckl. & Zeyh.	Linaceae	Root	Abdominal pains and diarrhea	[ 84]		
*Strychnos henningsii *Gilg ^†^	Loganiaceae	Root/bark	Diarrhea; pain and arthritis	[ 84,138,139]		Alkaloid (O-acetylretuline) and triterpenoid (Friedelin) [140]
*Strychnos spinosa *Lam.^†^	Loganiaceae	Root/bark/leaves	Diarrhea, dysentery, stomach ache and body pain	[ 52,94,141]		Secoiridoid glucosides
*Strychnos potatorum *L.f.	Loganiaceae	Roots/leaves	Stomach ache, toothache	[ 94]		
*Tapinanthus bangwensis *(Engl. & K.Krause)*Danser*	Loranthaceae	Leaves	Stomach pain	[ 52]		
*Punica granatum *L. ^†^	Lythraceae	Roots/fruit rind	Diarrhea and dysentery	[ 28]		Tannins, flavonoids, alkaloids, triterpenoids and sterols [142]
*Dissotis princeps *(Kunth) Triana ^†^	Melastomataceae	Leaves	Diarrhea and dysentery	[ 78]		
*Bombax* *buonopozense * ^†^	Malvaceae.	Bark/root	Diarrhea and dysentery and chest pain	[ 52]	Methanolic extract tested against castor oil-induced diarrhea [143]	Alkaloids, flavonoids, tannins, saponins, terpenoids, steroids, phlobatannins, anthraquinones and carbohydrates [144]
*Adansonia digitata *L.^†^	Malvaceae	Bark/leaves/fruit	Diarrhea, stomach pain	[ 52]	Hot plate, human diarrhea [86]	
*Dombeya rotundifolia *Planch.	Malvaceae	Root/bark/wood	Diarrhea and pain	[ 79]		
*Grewia bicolor *Juss.	Malvaceae	Roots/bark/leaves	Chronic diarrhea	[ 97]		
*Sida alba *L.	Malvaceae		Diarrhea and dysentery	[ 57]		
*Hibiscus aethiopicus*	Malvaceae	Root	Painful swollen joints	[ 84]		
*Hibiscus fuscus *Garcke	Malvaceae	Leaves	Chronic diarrhea	[ 97]		
*Azadirachta indica *^†^, *Azadirachta indica *A.Juss.	Meliaceae	Leaves, roots/bark/leaves	Stomach pain Headache, analgesic & anti-inflammatory	[ 52,94]	Tail flick, writhing-opioid [145] and non-opioid	Nimbin, nimbinin, nimbidin and azadirachtin
*Ekebergia capensis * ^†^	Meliaceae	Root	Dysentery, diarrhea, intestinal complaints; chest pains and headache	[ 79,84]		
*Ekebergia benguelensis Welw. ex C.DC.*	Meliaceae	Roots/bark leaves	Stomach ache	[ 94]		
*Khaya senegalensis *(Ders.) A. Juss.	Meliaceae	Stem bark	Diarrhea,	[ 61]		
*Melia azadirachta*	Meliaceae	Root/stem/leaves/fruit seed	Abdominal pains	[ 84]	Effective writhing test not carageenan [146]	Triterpenoids, steroids, gedunin, limonoids, coumarins, flavonoids
*Trichilia emetica; *relatedspp ^†^	Meliaceae	Bark, leavesand seeds	Stomach and intestinal pains and rheumatism	[ 84]		Tannin and resins
*Turraea floribunda*	Meliaceae	Roots	Painful joints, rheumatism	[ 84]		Limonoids
*Melianthus comosus Hochst. * ^†^	Melianthaceae	Root and leaves	Dyspepsia, diarrhea; rheumatism and painful feet	[ 84]		Triterpenoids and bufadinolides
*Albertisia delagoensis *(N.E.Br.) Forman ^†^	Menispermaceae	Roots	Diarrhea, dysentery, colic	[ 147]		Bisbenzylisoquinoline alkaloids [147]
*Antizoma angustifolia *(Burch.) Miers ex Harv. ^†^	Menispermaceae	Roots	Diarrhea, dysentery, colic	[ 147]		
*Cissampelos mucronata *A.Rich*.*	Menispermaceae	Roots	Stomach pain	[ 52]	Sedative effects [87]	Alkaloids, triterpenes, tannins, sterols, carbohydrates, glycosides, and flavonoids
*Cissampelos pareira L.*	Menispermaceae.	Roots	Stomach ache	[ 94]		
*Cissampelos capensis *(L.f.) Diels	Menispermaceae	Rhizome	Dysentery	[ 62,147]		
*Cissampelos torulosa *E.Mey. ex Harv. & Sond.	Menispermaceae	Rhizome	Diarrhea and dysentery	[ 57]		
*Ficus gnapalocarpa*	Moraceae	Leaves	Chest pain	[ 52]		
*Ficus vogelii*	Moraceae	Roots	Body pain	[ 52]		
*Psidium guajava *L. ^†^	Myrtaceae	Roots/bark/leaves	Diarrhea, dysentery	[ 5,88,94,97]		
*Syzygium guineense *(Willd.) DC.	Myrtaceae	Bark	Chronic diarrhea	[ 97]		
*Syzygium cordatum *Hochst. ex Krauss	Myrtaceae	Bark/leaves	Diarrhea, dysentery	[ 79]		
*Ximenia caffra Sond.*	Olacaceae	Roots/leaves	Stomach ache,	[ 94]		
*Olea europaea subsp.* *Africana.*	Olacaceae	Fruit	Dysentery, diarrhea	[ 5]		
*Papaver somniferum *L.	Papaveraceae		Narcotic; analgesic	[ 70]	Known	Alkaloid (opium poppy) [70]
*Harpagophytum procumbens*	Pedaliaceae		Anti-inflammatory; anti-rheumatic	[ 70]	Standard pain test (HP, PE). Used in humans [22,148]	Coumarins and phenolic glycosides [70]
*Ceratotheca triloba *(Bernh.) Hook.f.	Pedaliaceae	Leaves	Diarrhea, gastrointestinal cramps	[ 125]		
*Clutia spp*	Peraceae	Leaves	Painful joints, back and rheumatism	[ 84]		
*Phytolacca americana*	Phytolaccaceae	Roots/leaves/Fruit	Diarrhea, rheumatism	[ 84]		Triterpenoids and saponin
*Antidesma venosum *E.Mey. ex Tul.	Phyllanthaceae	Roots	Chronic diarrhea	[ 97]		
*Bridelia micrantha * ^†^	Phyllanthaceae	Root/stem bark/leaves	Diarrhea, epigastric pain, toothache	[ 84]	Castor-oil, charcol meal anti-motility in rats [149]	Friedelin, epi-friedelin, gallic acid, anthocyanidin, taraxerol, taraxerone and caffeic acid.
*Pseudolachnostylis maprouneifolia Pax*	Phyllanthaceae	Roots/bark/leaves	Diarrhea; stabbing sensations	[ 94]		
*Pittosporum viridiflorum sims*	Pittosporaceae	Stem bark	Stomach and abdominal, chest and back pains	[ 84]		Saponins and sesquiterpenoids
*Scoparia dulcis L.*	Plantaginaceae	Stalk	Severe chest pain	[ 52]		
*Plantago major L.*	Plantaginaceae	Leaves	Diarrhea and pain	[ 104]	Castor oil-induced diarrhea, anti-motility assay	Tannins, flavonoids, triterpenes, unsaturated sterols, proteins, amino acids, carbohydrates and lactones
*Plumbago auriculata*	Plumbaginaceae	Root/leaves	Painful joints, fractures	[ 84]		Naphthoquinone and plumbagin
*Plumbago zeylanica L.*	Plumbaginaceae	Root, leaves	Diarrhea, headaches	[ 52]		
*Imperata cylindrical*	Poaceae	Root	Darrhea, dysentery and pain	[ 52]		
*Cenchrus ciliaris L.*	Poaceae	Rhizome	Bovine viral diarrhea, pain	[ 84,150]		
*Protea simplex * ^†^	Poaceae	Root bark	Dysentery, diarrhea and stomach pain	[ 78]		
*Protea welwitschii *Engl.	Poaceae	Root bark	Dysentery and diarrhea	[ 53]		
*Securidaca longipedunculata *Fres*.*	Polygalaceae	Roots, leaves	Stomachache, headache and toothache	[ 94]		
*Rumex obtusifolius *L.	Polygalaceae	Leaves	Diarrhea	[ 5]		
*Rapanea melanophloeos* (L.)Mez	Primulaceae	Bark/root	Stomach pain and diarrhea	[ 84,136]		Tannin, triterpenoids and saponin
*Berchemia zeyheri*	Rhamnaceae	Stem bark	Backache	[ 84]		Pentahydroxychachones
*Helinus intergrifolius*	Rhamnaceae	Root	Painful joints and backache	[ 84]		Scyllitol, tannins and saponin
*Prunus africana *Hook. f.	Rosaceae	Roots, bark/fruits	Diarrhea, abdominal ailments, intercostal-pain	[ 5,84]		Amygdalin, friedelin, hydrocyanic, ursolic acids, sterols, cyanogenic glycosides and saponins
*Rubus rigidus *spp	Rosaceae	Roots	Diarrhea, dysentery and toothache	[ 84]		Tannins and pyragallol
*Breonadia salicina *(Vahl) Hepper & J.R.I.Wood ^†^	Rubiaceae	Bark	Diarrhea, bloody stool and colic	[ 151]		
*Catunaregam spinosa *(Thunb.) Tirveng*.*	Rubiaceae	Roots/bark	Stomach ache	[ 94]		
*Crossopteryx febrifuga* (G.Don) Benth. ^†^	Rubiaceae	Roots/bark	Diarrhea, dysentery, stomach ache	[ 94]		
*Gardenia erubescens*	Rubiaceae	Roots	Headache	[ 52]		Polyphenols
*Mitragyna inermis (*Willd.) Kuntze	Rubiaceae	Stem/leaves	Body pain, diarrhea	[ 52]	Anti-motility effect in rat ileum [152]	Triterpenoid saponins and alkaloids
*Nauclea latifolia *(Sm.*) E.A.Bruce *^†^	Rubiaceae	Root/bark	Diarrhea/dysentery/pain/inflammation	[ 52,153,154,155]	Chemical pain, hot-plate and tail flick; opioid, purinergic, GABAergic; diarrhea + antimotility tests [153,156]	Polyphenolics, flavonoids, triterpenes and sterols [107,157]
*Pentanisia prunelloides (Klotzsch) Walp. * ^†^	Rubiaceae	Roots/bulb/leaves	Diarrhea, abdominal and chest-pains and rheumatism	[ 5,84]		
*Psychotria capensis *(Eckl.) Schönland			Diarrhea	[ 5]		
*Rubia petiolaris *DC*.*	Rubiaceae	Root	Diarrhea and dysentery	[ 28]		
*Agathosma betulina *(P.J.Bergius) Pillans ^†^	Rutaceae		Cholera, diarrhea and dysentery and antispasmodic	[ 158]		
*Agathosma crenulata *(L.) Pillans ^†^	Rutaceae		Cholera, diarrhea and dysentery, and antispasmodic	[ 158]		
*Clausena anisata *(Willd.) Hook.f. ex Benth.	Rutaceae	Root/stem/leaves	Abdominal pain toothache and rheumatism	[ 84,131]		Terpenoids, alkaloids, coumarins and limonoids
*Citrus aurantifolia *(Christm.) Swingle	Rutaceae	Root	Stomach pain	[ 52]		Lime
*Zanthoxylum zanthoxyloides *(Lam.)Zepern. & Timler*or (Fagara Zanthoxyloides) *^†^	Rutaceae	Stem bark/leaves	Diarrhea, dyspepsia, toothache, abdominal pain	[ 52,138]	Tested against pain [159]	Alkaloids, flavonoids and coumarins
*Zanthoxylum chalybeum* *Engl.*	Rutaceae	Roots/bark /leaves	Stomachache, headache, and toothache	[ 94]		
*Ziziphus mucronata *Willd*.*^ †^	Rutaceae	Roots/bark/leaves	Stomach ache, diarrhea, dysentery and chest pains	[ 79,94]		
*Ziziphus Zeyheriana *Sond. ^†^	Rutaceae	Roots	Diarrhea and general gastrointestinal ailments	[ 53]		
*Flacourtia indica *(Burm.f.) Merr.	Salicaceae	Roots/leaves	Stomach ache	[ 94]		
*Hippobromus pauciflorus *Radlk*.*	Sapindaceae	Root/bark/leaves	Diarrhea, dysentery and headache,	[ 5]		
*Paullinia pinnata L.*	Sapindaceae	Leaves	Diarrhea, body pain	[ 52]		Triterpenoids and flavone glycosides
*Zanha africana *(Radlk.)Exell	Sapindaceae	Roots/bark	Stomachache, headache	[ 94]		
*Manilkara concolor Harv. * ^†^	Sapotaceae	Root/stem bark	Diarrhea; joint and back pain	[ 84]		
*Datura suaveolens*	Solanaceae	Leaves	Internal inflammation and body pain	[ 52]	GABA receptors [160]	Scopolamine (hyoscine), ropine, hyoscyamine, and other alkaloids
*Physalis peruviana L. *	Solanaceae	Leaves	Abdominal ailment	[ 5]		
*Solanum spp *	Solanaceae	Root/bark /leaves/fruits	Abdominal pain, toothache, rheumatism	[ 84]		Alkaloids, vitamin C and carotene
*Solanum aculeastrum* Dunal	Solanaceae	Roots/leaves/fruits	Dysentery, diarrhea	[ 5]		
*Solanum incanum* L.	Solanaceae		Stomach ache,	[ 94]		
*Pouzolzia mixta *Solms ^†^	Urticaceae	Roots/leaves	Diarrhea and dysentery	[ 57,79]		
*Lippia multifora *Moldenke	Verbenaceae	Leaves	Chest and body pain	[ 52]		
*Lippia javanica *(Burm.f.) Spreng. ^†^	Verbenaceae	Root/stem/leaves	Diarrhea, dysentery, chest pain, rheumatism	[ 84,117]		Pentacyclic triterpenoids, essential oil, amino acids, and other acids
*Bulbine abyssinica *^†^	Xanthorrhoeaceae	Tubers/leaves	Diarrhea; rheumatism	[ 5]		
*Bulbine asphodeloides * ^†^	Xanthorrhoeaceae	Tuber/leaves	Dysentery and diarrhea	[ 84]		
*Bulbine latifolia * ^†^	Xanthorrhoeaceae	Leaves	Dysentery diarrhea and pain	[ 84,102]		

Blanks: Unknown or the existence of this information was not determined through literature search. However, for the majority the listed plant species the information is not known. ^†^ The extracts of these plant species are recommended for high throughput screening for bioactive agents to treat gastrointestinal (GI) disorders such as diarrhea, inflammation and chronic pain. These plants have potential for novel complementary drugs against GI disorders presenting with diarrhea, dysentery, and chronic pain and hence complementary usage with acupuncture and western medications.

**Table 3:** Summary Table demonstrating the overlap and differences of plant families used TAHM (veterinary and human care) and TCHM (veterinary care).

Family name TAHM (Veterinary)	Number of appearance	TAHM (human)	Number of appearances	TCHM (veterinary)	Number of appearances
		Aizoaceae	2		
Amaranthaceae	2	Amaranthaceae	4	Amaranthaceae	2
Amaryllidaceae	1	Amaryllidaceae	4		
Anacardiaceae	5	Anacardiaceae	11		
Annonaceae	2	Annonaceae	3		
Apiaceae	1	Apiaceae	4	Apiaceae	1
Aponynaceae	5	Apocynaceae	7		
Araliaceae	1	Araceae	1		
Asparagaceae	3	Asparagaceae	7		
Asteraceae	1				
		Balanophoraceae	1		
				Berberidaceae	3
		Bignoniaceae	3		
Boraginaceae	1				
Brassicaceae	1	Brassicaceae	2		
Campanulaceae	1			Campanulaceae	1
		Capparaceae	2		
		Caprifoliaceae	1	Caprifoliaceae	2
Cannabaceae	1	Cannabaceae	1		
		Canellaceae	1		
Celastraceae	2	Celastraceae	3		
Cesalpiniaceae	1				
		Chrysobalanaceae	1		
		Clusiaceae	4		
Combretaceae	3	Combretaceae	11		
		Commelinaceae	1		
Compositae	9	Compositae	20	Compositae	4
		Convolvulaceae	2		
		Crassulaceae.	1	Crassulaceae	2
Cucurbitaceae	2				
Cupressaceae	1				
		Curtisiaceae	1		
				Dipsacaceae	1
Dryopteridaceae	1				
Ebenaceae	1	Ebenaceae	2		
				Ericaceae	3
Euphorbiaceae	2	Euphorbiaceae	5	Euphorbiaceae	1
				Gentianaceae	3
Geraniaceae	3	Geraniaceae	5		
		Gunneraceae	1		
		Hydnoraceae	1		
		Hymenocardiaceae	1		
Hypericaceae	3	Hypericaceae	2		
		Hypoxidaceae	2		
		Icacinaceae	1		
Iridaceae	2	Iridaceae	2		
Lamiaceae	6	Lamiaceae	8	Lamiaceae	2
		Lauraceae	2		
Leguminosae	24	Leguminosae	47	Leguminosae	5
		Linaceae	1		
Loganiaceae	1	Loganiaceae	4		
Loranthaceae	1	Loranthaceae	2		
Lythraceae	1	Lythraceae	1		
		Melastomataceae	1		
Malvaceae	5	Malvaceae	7		
Meliaceae	2	Meliaceae	8		
		Melianthaceae	1		
Menispermaceae	5	Menispermaceae	6	Menispermaceae	1
		Moraceae	2	Moraceae	1
Musaceae	2				
Myrtaceae	4	Myrtaceae	6		
Nymphaeaceae	1				
Olacaceae	1	Olacaceae	2		
Orobanchaceae	1				
		Papaveraceae	1	Papaveraceae	5
		Pedaliaceae	2	Pedaliaceae	1
Peraceae	1	Peraceae	1		
Phytolaccaceae	3				
Piperaceae	1				
		Phyllanthaceae	3		
		Phytolaccaceae	1		
Pittosporaceae	1	Pittosporaceae	1		
		Plantaginaceae	2	Plantaginaceae	1
Plumbaginaceae	1	Plumbaginaceae	2		
Poaceae	6	Poaceae	4		
		Polygalaceae	2	Polygonaceae	2
		Primulaceae	1		
		Rhamnaceae	2		
Ramalinaceae	1				
Rosaceae	1	Rosaceae	3		
				Ranunculaceae	10
Rubiaceae	3	Rubiaceae	11	Rubiaceae	1
Rutaceae	1	Rutaceae	9		
		Salicaceae	2		
Sapindaceae	1	Sapindaceae	3		
Sapotaceae	1	Sapotaceae	1		
				Scrophulariaceae	2
Solanaceae	3	Solanaceae	7	Solanaceae	4
Sterculiaceae	1				
		Thymelaeceae	1	Thymelaeaceae	1
				Umbelliferae	1
Urticaceae	2	Urticaceae	1		
Verbenaceae	1	Verbenaceae	2	Verbenaceae	1
Vitaceae	1				
		Xanthorrhoeaceae	3		
				Zingiberaceae	3
Zygophyllaceae	1				

TAHM = traditional African herbal medicine (both veterinary and human care). TCHM = traditional Chinese herbal medicine (veterinary care only). The number of appearances does not represent the actual appearances in literature but the actual appearances in references cited here.

**Table 4:** An inventory of Chinese herbal preparations used to treat animal gastrointestinal pain and diarrhea.

Species	Family	Part used	China	Tests (W, T)	Compounds/class
*Achyranthes bidentata*	Amaranthaceae		Inflammation, arthritis and pain	[ 161]	Triterpenoid and saponin
*Achyranthes aspera* L.	Amaranthaceae	Whole plant	Dysentery, inflammation	[ 161]	
*Bupleurum chinense *DC	Apiaceae		Diarrhea due to spleen deficiency	[ 161]	Saponins, volatile oils, oleic/palmitic/linoleic acids, and alpha-spinasterol
*Berberis julianae* Schneid	Berberidaceae	Roots	Dysentery	[ 162]	
*Epimedium brevicornum, * *E. pubescens, E wushanense and * *E. Koreanum*	Berberidaceae		Rheumatism	[ 161] Compound formulas	Volatile oils, flavonoids, anthraquinones, polysaccharides, phytosterol, oleic/palmitic/linoleic acids
*Sinopodophyllum hexandrum*	Berberidaceae	Fruits and stems	Inflammation	[ 162]	
*Valeriana fauriei* Brig.	Caprifoliaceae (Plant list) Valerianaceae	Roots	Inflammation	[ 162]	
*Lonicera japonica *var*. chinensis (*P. Watson*) Baker *	Caprifoliaceae	Various preparations and doses for treating animals and fish	Dysentery and inflammation	[ 161]	Triterpenoids saponins flavonoids and tannins
*Saussurea epilobioides* Maxim. var. cana Hand.-Mazz.	Compositae	Herbs	Pain	[ 162]	
*Saussurea medusa* Maxim	Compositae	Herbs	Inflammation	[ 162]	
*Senecio diversipinnus* Ling.	Compositae	Aerial parts	Dysentery	[ 162]	
*Soroseris hookeriana* (C.B.Clarke)	Compositae	Herbs	Inflammation and pain	[ 162]	
*Codonopsis lanceolata (Siebold & Zucc.) Benth. & Hook.f. ex Trautv. *	Campanulaceae		Pain	[ 161]	Triterpene saponins, flavonoids, alkaloids, stigmasterol, syringarsinol α-spinasterol, hexadecane acid and succinic acid
*Rhodiola algida* (Ledeb) Fisch.et Mey. var. tangutica (Maxim.)	Crassulaceae	Roots	Pain and inflammation	[ 162]	
*Rhodiola kirilowii* (Regel)Maxim.	Crassulaceae	Flowers and leaves	Diarrhea and inflammation	[ 162]	
*Pterocephalus hookeri* (C.B.Clarke	Dipsacaceae	Herbs	Dysentery	[ 162]	
*Rhododendron anthopogonoides *Maxim.	Ericaceae	Flowers and leaves	Inflammation	[ 162]	
*Rhododendron latoucheaeo* Franch.	Ericaceae	Leaves	Inflammation	[ 162]	
*Euphorbia helioscopia *L.	Euphorbiaceae	Whole plant	Chronic diarrhea, dysentery and rheumatism	[ 161]	
*Gentiana dahurica Fisch.*	Gentianaceae	Flowers and roots	Inflammation	[ 162]	
*Gentiana straminea *Maxim*.*	Gentianaceae	Flowers	Diarrhea, inflammation	[ 162]	
*Gentianopsis grandis*	Gentianaceae	Herbs	Pain and diarrhea	[ 162]	
*Lamiophlomis rotata *(Benth. ex Hook.f.) Kudô	Lamiaceae (plant list) Labiatae	Aerial parts	Inflammation and pain	[ 162]	
*Scutellaria baicalensis*	Lamiaceae		Diarrhea anf joint pain and inflammation	[ 161]	Flavonoids, oleic/palmitic/benzoic acids, and sterols
*Abrus precatorius* L.	Leguminosae (plant list) Fabaceae	Root and leaf	Diarrhea, colic and pain	[ 161]	
*Acacia adsurgens *Maiden & Blakeley	Leguminosae (plant list) Mimosaceae	Stem and leaf	Headache (Australia)	[ 161]	
*Acacia ancistrocarpa *Maiden & Blakeley	Leguminosae (plant list) Mimosaceae	Stem and leaf	Headache	[ 161]	
*Acacia catechu *Willd.	Leguminosae (plant list Mimosaceae	Stem and leaf	Inflammation in china	[ 161]	
*Pueraria lobata*	Leguminosae		Diarrhea due to spleen deficiency	[ 161]	Isoflavones, arachidic acid, daucosterol and allantoin
*Tinospora sinensis *(Lour.) Merrill	Menispermaceae	Stem and root	Arthritis pain	[ 161]	
*Ficus tikoua *Bur*.*.	Moraceae	Stem	Rheumatic pain, acute gastroenteritis, dysentery	[ 161]	
*Eucalyptus pruinosa*Schau.	Myrtaceae (Australia only)	Inner bar	Pains, rheumatism, headache	[ 161]	
*Corydalis dasyptera* Maxim.	Papaveraceae	Herbs	Pain, stomach ailment	[ 162]	
*Corydalis melanochlora* Maxim.	Papaveraceae	Herbs	Pain, stomach ailment	[ 162]	
*Corydalis straminea* Maxim.	Papaveraceae	Herbs	Pain, stomach ailment	[ 162]	
*Hypecoum leptocarpum Hook.f.et Thoms.*	Papaveraceae	Herbs	Inflammatory, pain	[ 162]	
Papaver rhoeas L.	Papaveraceae	Flowers and herbs	Dysentery, pain,	[ 162]	
*Harpagophytum procumbes*	Pedaliaceae			[ 161]	Iridol plus phenolic glycosides (e.g., Harpagoside)
*Plantago major* L.	Plantaginaceae	Aerial parts	Dysentery,	[ 162]	
*Rheum palmatum *L.*, R. tanguticum, and R. officinale*	Polygonaceae		Stomach pain	[ 161]	Anthraquinones derivetives, tannins, sennoside, rhenosides, anthranol and anthrone
*Rheum glabricanle* G. San.	Polygonaceae	Roots and rhizomes	stomach pain,	[ 162]	
*Aconitum kongboense*	Ranunculaceae	Herbs	Inflammation, pain/ rheumatism	[ 162]	
*Aconitum tanguticum*	Ranunculaceae			[ 162]	
*Aconitum carmichaeli*	Ranunculaceae		rheumatism, abdominal pain, analgesic anti-inflammatory	[ 162]	Alkaloids, diterpenoids, aminophenols, hygenamine, coryneine and salsolinol
*Clematis glycinoides *DC.	Ranunculaceae	Leaf	Pains, rheumatism, headache	[ 162]	
*Coptis chinensis, C. deltoidea, * *C. Teeta*	Ranunculaceae		Diarrhea/dysentery	[ 161]	Isoquinoline alkaloids (e.g., berberine, epiberberine, columbamine), ferulic acid, chlorogenic acid
*Clematis chinensis* Osbeck	Ranunculaceae	Root	Rheumatoid arthritis, toothache, pain	[ 161]	
*Clematis tangutica* (Maxim.) Korsh.	Ranunculaceae	Stems and leaves	Dysentery dyspepsia	[ 162]	
*Delphinium caeruleum*	Ranunculaceae	Herbs	Dysentery	[ 162]	
*Delphinium candelabrum* var. monanthum	Ranunculaceae	Herbs	Dysentery	[ 162]	
*Morinda officinalis *How	Rubiaceae	Root	Rheumatism, leg pain, backsore	[ 161]	
*Pedicularis kansuensis* Maxim.	Scrophulariaceae	Flowers	Inflammation,	[ 162]	
*Pedicularis torta* Maxim.	Scrophulariaceae	Flowers	Inflammation,	[ 162]	
*Hyoscyamus niger L.*	Solanaceae	Seeds	Pain,	[ 162]	
*Scopolia japonica* Maxim.	Solanaceae	Rhizomes	Inflammation,	[ 162]	
*Solanum lyratum *Thunb.	Solanaceae	Whole plant	Rheumatism, headache,	[ 161]	
*Capsicum spp*	Solanaceae	Cayene pper and olive oil	Stomach illnesses	[ 161]	Capsaicin
*Stellera chamaejasme* L.	Thymelaeaceae	Roots	Inflammation,	[ 162]	
*Notopterygium incisum* Ting exH.T	Umbelliferae	Roots and rhizomes	Pain,	[ 162]	
*Verbena officinalis *L.	Verbenaceae	Whole plant	Dysentery, inflammation and pain	[ 161]	Apigenin, 4'-hydroxywogonin , verbenalin and hastatoside
*Amomum villosum *and *Amomum longiligulare*	Zingiberaceae	Comp formula	Diarrhea and pain	[ 161]	Saponins, flavonoids and volatile oils
*Curcuma longa *L.	Zingiberaceae	Tumeric	Inflammation,	[ 161]	
*Zingiber officinale*	Zingiberaceae	Root tuber (Ginger )	Inflammation and pain	[ 161]	

## 4. Future Perspectives

### 4.1. High Throughput Fractionation and Screening to Isolation, Characterization and Testing of Medicinal Components

Solvent fractionation, in combination with different chromatography techniques, can lead to separating the extract into a large number of fractions for bioassays. For example, with the Sepbox 2D HPLC [163] up to 576 fractions with a very high yield of pure compounds can be collected for subsequent biological screening. The Sepbox concept is based on a patented combination of HPLC and SPE (Solid Phase Extraction), using two-dimensional separation, one extract can be completely separated in vials or microtiter plates per day [163]. The production of such a large number of fractions makes it possible to screen a lot of different plant materials via high throughput screening (HTS), because many samples can be assayed in a short period of time. The basis of high throughput screening includes: reduction in size of test volumes, employment of high-density microwell plates, and automated processing. All of which make biotesting less labor-intensive, more reproducible, and less expensive as many routine tasks are performed by robots [45]. In essence, this strategy operates on detecting a specialized effect of a test compound or extract on receptors or enzymes (single-target specific bioassay) or on intact cells, isolated organs, or whole animals (multitarget functional bioassay) [45]. Conversely, HTS is based on a large number of samples, and researchers all over the world using ethnopharmacological approaches or chemical libraries build by elicitation of plant culture, combinatorial chemistry, combinatorial biosynthesis, engineering of biosynthetic pathways, induction of microbial secondary metabolism, and biotransformation [164].

In the past, lack of bioassays, time-consumption, and instability of bioassays hampered the search for “hits”. Recombinant DNA technologies have facilitated the development of cell-based assays. Eukaryotic cells can be engineered to produce a specific gene product in response to a stimulus, and reporter genes are frequently used as indicators of transcriptional activity or activation of particular signaling pathways within the cell [45]. These techniques are needed to screen and test bioactive components from African botanical extracts that have been documented or shown to have therapeutic effects against pain and gastrointestinal disorders. 

Validation of preliminary hits by mammalian animal models is slow and expensive. The use of the zebrafish-based assays combine the advantage of HTS assays, compared to mammalian models, with the outcome being greater relevance to humans. The primary advantages of zebrafish for drug discovery include their high genetic, physiologic, and pharmacologic similarity with humans, as well as their small size, optical transparency, rapid development, and large numbers of embryos and larvae (which are the primary system for experimental analysis). Because of their small size (1 to 5 mm), zebrafish embryos and larvae are compatible with microtiter plates for screening, thereby requiring only microgram amounts of each extract, fraction, or compound to be tested. Because of the high fecundity of zebrafish, large numbers of embryos and larvae can be produced and analyzed in a more cost-effective manner than, for example, mice and rats. Combined, these features define zebrafish as an ideal *in vivo* model for the systematic identification of bioactive natural products with therapeutic potential [165]). 

Bioactivity screening in zebrafish embryos of over 80 east African medicinal plants yielded two methanolic extracts from *Oxygonum sinuatum* (Meisn.) Dammer (Polygonaceae) and *Plectranthus barbatus* Andrews (Lamiaceae), which inhibited intersegmental vessel (ISV) outgrowth in *fli-1*: enhanced green fluorescent protein (EGFP) embryos in a dose-dependent manner. Zebrafish bioassay-guided fractionation identified the active components of these plants as emodin (an inhibitor of the protein kinase CK2) and coleon A lactone (a rare abietane diterpenoid with no previously described bioactivity). Both emodin and coleon A lactone inhibited mammalian endothelial cell proliferation, migration, and tube formation *in vitro*, as well as angiogenesis in the chick chorioallantoic membrane (CAM) assay [166]. Few studies indicate zebra fish can be employed in studies of pain [167] and gastrointestinal disorders [168]. Clearly zebra fish should be considered when choosing models for testing the bioactivity of extracts from African botanical remedies of pain and gastrointestinal disorders. 

Whether natural product drug discovery programs should rely on wild plants collected “randomly” from the natural environment, or whether they should also include plants collected on the basis of use in traditional medicine, remains an open question. However, in this review, we emphasize the need to use TAHM documented practices for choosing plant species for screening. As indicated earlier, this view is supported by the analysis conducted by [20] showing that therapeutic uses of about 80% of 122 plant-derived drugs correspond to their original ethnopharmacological role. Similarly, a study conducted in Vietnam and Laos by Gyllenhaal and colleagues [169] suggests that ethnomedical uses may contribute to a higher rate of activity in drug discovery screening. A random bioassay-guided fractionation of 7,500 traditional medicinal plant extracts from South Africa to identify fractions with anticancer activity and active constituents showed that 68% of these plant species—which were hits in the screening program—were reported to be used medicinally. Based on this data, it appears that unrelated medicinal use of the source plants may serve as an initial guide to selection of plants for anticancer screening [117]. This supports the indigenous knowledge of using an individual extract for treating more than one illness.

Bioassay-guided fractionation of the dichloromethane root bark extract of *Entada abyssinica A.Rich.*, a plant used by traditional healers in Uganda for the treatment of sleeping sickness, led to the isolation of a diastereoisomer of the clerodane type diterpene kolavenol. It showed a trypanocidal activity with an IC_50_ value of 2.5 µg/mL (8.6 µM) against *Trypanosoma brucei rhodesiense*, the causative agent of the acute form of human African trypanosomiasis [170]. In this case, a dereplication process or a partially performed dereplication process might have excluded the extract from further investigation, but as reported, a diastereomer of the already described kolavenol was identified. 

Extracts from 11 West African plants traditionally used to treat malaria in Ghana were tested against both the chloroquine-sensitive strain (PoW) and the chloroquine-resistant clone Dd2 of *Plasmodium falciparum. *Due to the promising *in vitro *activity of the lipophilic extract [IC_50_: 10.5 μg/mL (PoW); 13.1 μg/mL (Dd2)], *Microglossa pyrifolia *(Lam.) Kuntze was chosen for further phytochemical investigation. Bioassay-guided fractionation of the aerial parts of *M. pyrifolia* revealed the fractions eluting from a RP-18 column with 80% to 100% MeOH to be most active with IC_50_ values ranging from 2.5 to 18.7 μg/mL against PoW and a Dd2 starins of *Plasmodium falciparum. *From active fractions, 13 compounds were isolated and their structures were established on the basis of spectroscopic methods. The two diterpenes *E*-phytol [IC_50_: 8.5 μm (PoW); 11.5 μm (Dd2)], and 6*E-*geranylgeraniol-19-oic acid [IC_50_: 12.9 μm (PoW); 15.6 μm (Dd2)] proved to be the most active constituents [171]. Normally, fractions showing high activity (or higher activity than others) are further bioactivity-guided fractionated until the bioactive compound/s is/are obtained in pure form. In cases in which—from a bioactive fraction—the major components were isolated and afterwards the single compounds were measured, the major bioactive compound could be overseen. This is explainable, if the major bioactive compound is not the most abundant in the chromatogram (perhaps not UV active), or no quantitation of the isolated compounds is performed, or no recombination of the isolated compounds is performed. 

The temporary immersion system (TIS) for the *in vitro* production of bioactive compounds (e.g., in Harpagophytum (Devil’s Claw)), might be an option for fast and easier production of bioactive lead compounds [172]. *Harpagophytum procumbens* DC. is a perennial herbaceous plant growing in South and Tropical Africa, especially in the Kalahari Desert and in the Namibian steppes. The dried aqueous extract is known for its anti-inflammatory effects [173]. In this study, the immersion time of TIS cultures (200 mL) was 1 h per day over 4 months. The biomass produced consisted mainly of normal plants with rooted leafy shoots, together with some partially differentiated callus. The total iridoid levels in root, stem, and leaf tissues of TIS grown plants were similar to that of two-year old glasshouse grown whole plants [172]. 

To summarize, research suggests that botanical remedies used in TAHM have components with medicinal activities. Apparently, plant species used in TAHM to treat pain, inflammation, and gastrointestinal disorders shown here have never been subjected to HTS screening. Based on folklore description and scant evidence to support to the presence of components with analgesic and anti-motility effects, there is the need to use HTS to screen and identify neuroactive compounds as well as anti-inflammatory compounds. Although high throughput screening requires a state-of-the art infrastructure (which is not available in many African institutions) it is still possible through collaborative research partnerships between African and western countries and China. 

### 4.2. Bioactivity Testing for Analgesic and Anti-Diarrheal Fractions and Compounds

The majority of studies done to verify analgesic and anti-inflammatory effects of extracts used in TAHM use chemical and thermal agents to induce pain and inflammation [95,129,174,175]. The presence of anti-diarrheal agents is determined using anti-motility and anti-secretory assays in intact animals, isolated tissues, and cells [114,115,153,176]. The goal of the vast majority of these studies is to determine the basis for supporting folklore practices [23]. There is a critical need to investigate the analgesic potential of plant extracts through identification of bioactive compounds, their mechanisms of action, efficacy, and toxicity in search for new pain-relievers [19,173,175,177,178,179] and anti-diarrheal drugs [114,115,136,176,178,180]. This is necessary to verify the effectiveness of botanical therapies and to provide solid evidence for safety. In addition, contemporary analgesics, such as opiates and nonsteroidal anti-inflammatory drugs, are often not suitable in all cases, particularly chronic pain due potency, side effects, and tolerance (e.g., opiates and cannabinoids) [181,182,183]. Medicinal plants are known to be an important source of new chemical substances with potential therapeutic effects [2,18,19,20,178,184,185]. Based on the advances being made in pain research, determination of antinociceptive profiles of crude extracts and semipurified extracts should be done as means for rapid screening specific tests as highlighted below:

#### 4.2.1. Opioid Receptors

Opioid receptors are well known targets of natural and synthetic anti-nociceptive agents. Opium alkaloids are among the plant metabolites with anti-nociceptive effects. In the gastrointestinal tract, opioid receptor agonists (such as morphine and loperamide) exhibit anti-nociceptive and anti-motility effects as well as anti-diarrheal effects. Botanical extracts with analgesic and anti-diarrheal effects should be screened for activity against opioid receptors to determine the presence of non-opiate compounds [115,181]. Opiates such as loperamide, cannabinoids, and non-opiate preparations with antinociceptive effects against painful gastrointestinal disorders reduce motility and have anti-diarrheal effects. Unfortunately, the only drugs available that rapidly shorten the duration of diarrhea and alleviate gastrointestinal pain are opiates. Opiates, cause constipation, drowsiness, and are addictive in humans [178,181]. This is an important gap in the treatment of diarrheal illnesses and the accompanying pain and stress found in both veterinary and human patients. This suggests that screening plant species with analgesic properties could reveal new anti-motility agents, presumably flavanone, flavonoids and triterpenoids [19,34,114,118,148,178,186,187,188]. 

#### 4.2.2. Cannabinoid Receptors

Cannabinoid CB(2) receptors are considered to be targets for novel analgesia [189] drugs. In the gut, CB2 receptors are also involved in visceral pain, inflammation, and motility disturbances [183]. It is unclear whether non-addictive cannabinoids exist among phytochemical compounds or if HTPS of TAHM could reveal new, non-addictive therapies that target CB(2) receptors [182,183].

#### 4.2.3. Transient Receptor Potential (Vanilloid) Receptors

There is evidence to suggest that TRPV1 receptor antagonists are promising non-narcotic, new analgesic medications that can be used to block painful sensations and can be administered orally [178]. Botanical extracts are potential sources of novel flavonoids and other compounds having potential as specific TRP antagonists as shown in recent extensive reviews [17,178,186,190].

#### 4.2.4. Purinergic Receptors

Novel research suggests that adenosine triphosphate (ATP) plays a crucial role in immune system-neuronal nociceptive signals transmission. P2X, the purinergic family of receptors, are crucial targets for inhibiting microglia–neuron interactions for the management of neuropathic pain and inflammation [177,191,192,193,194]. Recent bioactivity tests have demonstrated that Chinese herbal preparations and derived compounds (e.g., tetramethylpyrazine, sodium ferulate, puerarin, and lappaconitine) inhibit nociception mediated by P2X(3) and/or P2X(2/3) receptors, and suggest that botanical extracts are potential sources of compounds that can block the various families of P2 receptors of pain [175,195]. The analgesic effect of lappaconitine involves a decrease in expression and sensitization of the P2X(3) receptors of the rat DRG neurons [195]. Apparently, none of the African herbal extracts have been tested for inhibiting macrophage and neuronal ATP receptors, indicating an urgent need to do so. It is conceivable to assume that novel, selective, and non-selective P2X antagonists will be found through high throughput bioactivity guided screenings of African herbal and plant remedies with analgesic effects (indicated in Table 1 and Table 2), and that these agents will benefit veterinary patients. 

#### 4.2.5. Metabotropic Glutamate Receptors

Metabotropic glutamate receptors are implicated in inflammatory and neuropathic pain conditions in the gut and whole body in general [196,197]. There is evidence to suggest that medicinal plants, having antinociceptive compounds, may exert their effect via glutamate receptors [198] indicating another type of candidate target receptor for screening antinociceptive from African plant extracts with reported folklore use against gastrointestinal pain.

#### 4.2.6. Gamma-Aminobutyric Acid (GABA) Receptors

Although the impact of GABA receptors in pain and pain management is not well understood, several lines of evidence suggest that GABA receptors are important targets of pain management. First, the ion channel GABA(A)-receptor activation is involved in spinal pain signaling during persistent inflammation or inflammatory hyperalgesia [199]. Second, it has been demonstrated that activating the G-protein-coupled GABA(B) receptors with baclofen inhibits visceral anti-nociceptive effects in rats [200]. Third, there is evidence suggesting that herbal extracts from African botanical remedies have compounds that affect GABA neurotransmission [201,202]. This indicates that such extracts and derived compounds, especially those affecting the aforementioned GABA receptors have the potential be used as medication of chronic pain. In our studies of a traditional African anti-diarrhea remedy, the aqueous *G. buchananii* stem bark extract, we have found that the extract has antinociceptive effects [116] and affects GABA receptors [115], suggesting that *G. buchananii* should be included among plant species for screening against GABA receptors. Fifth, novel GABA receptor modulators include flavonoids, terpenoids, phenols, and polyacetylenic alcohols [201,202,203]. When taken together, this information shows that there is potential for using botanical extracts and derivative components that affect GABA signaling as individual or adjunctive therapies of animal patients. 

In contrast to human traditional African medicine, ethnoveterinary practices are not so well developed in African veterinary medicine, and still lack general acceptance [204]. Clinical trials, for instance trials showing the efficacy of extracts of *Aloe secundiflora* against *Salmonella gallinarum* in chicken [205], and *Vernonia amygdalina* against helminth in dogs [206], are needed for these animal illnesses of economic importance. Case reports and observational studies conducted by veterinarians/animal scientists are critically needed to promote TAM for veterinary patients.

Population growth associated with the increased need for herb and plant products as medication and remedies in native countries and abroad, urbanization, modern faming and mining are rapidly leading to the depletion of habitat and loss of medicinal plants [207,208]. Re-aforastation programs at community level, growth of native medicinal plants, agricultural transformation including farming TAM plants, growing medicinal plants back yards and afforestation projects should be given priority. 

## 5. Conclusions

To conclude, Africa has tremendous untapped resources of natural medications for gastrointestinal ailments, pain, and inflammation. Prioritized, extensive HTS and bioactivity testing are required to identify the most efficacious and safe preparations, and their mechanisms of action. This will lay a solid foundation for the development of commercial products that can be integrated with acupuncture and western medicine to treat veterinary and human patients against chronic pain, gastrointestinal disorders. This is a critical time for conservation of medicinal plants before we lose them. 
